# Proteomic Profiles of Adipose and Liver Tissues from an Animal Model of Metabolic Syndrome Fed Purple Vegetables

**DOI:** 10.3390/nu10040456

**Published:** 2018-04-06

**Authors:** Hala M Ayoub, Mary Ruth McDonald, James Alan Sullivan, Rong Tsao, Kelly A Meckling

**Affiliations:** 1 Department of Human Health and Nutrition Sciences, University of Guelph, Guelph, ON N1G 2W1, Canada; hayoub@uoguelph.ca; 2 Department of Plant Agriculture, University of Guelph, Guelph, ON N1G 2W1, Canada; mrmcdona@uoguelph.ca (M.R.M.); asulliva@uoguelph.ca (J.A.S.); 3 Guelph Research & Development Centre, Agriculture and Agri-Food Canada, Guelph, ON N1G 5C9, Canada; Rong.Cao@agr.gc.ca

**Keywords:** obesity, hypertension, insulin resistance, high fat diet, carrots, potatoes, proteomic analyses

## Abstract

Metabolic Syndrome (MetS) is a complex disorder that predisposes an individual to Cardiovascular Diseases and type 2 Diabetes Mellitus. Proteomics and bioinformatics have proven to be an effective tool to study complex diseases and mechanisms of action of nutrients. We previously showed that substitution of the majority of carbohydrate in a high fat diet by purple potatoes (PP) or purple carrots (PC) improved insulin sensitivity and hypertension in an animal model of MetS (obese Zucker rats) compared to a control sucrose-rich diet. In the current study, we used TMT 10plex mass tag combined with LC-MS/MS technique to study proteomic modulation in the liver (*n* = 3 samples/diet) and adipose tissue (*n* = 3 samples/diet) of high fat diet-fed rats with or without substituting sucrose for purple vegetables, followed by functional enrichment analysis, in an attempt to elucidate potential molecular mechanisms responsible for the phenotypic changes seen with purple vegetable feeding. Protein folding, lipid metabolism and cholesterol efflux were identified as the main modulated biological themes in adipose tissue, whereas lipid metabolism, carbohydrate metabolism and oxidative stress were the main modulated themes in liver. We propose that enhanced protein folding, increased cholesterol efflux and higher free fatty acid (FFA) re-esterification are mechanisms by which PP and PC positively modulate MetS pathologies in adipose tissue, whereas, decreased *de novo* lipogenesis, oxidative stress and FFA uptake, are responsible for the beneficial effects in liver. In conclusion, we provide molecular evidence for the reported metabolic health benefits of purple carrots and potatoes and validate that these vegetables are good choices to replace other simple carbohydrate sources for better metabolic health.

## 1. Introduction

Metabolic Syndrome (MetS) is a complex disorder that predisposes an individual to type 2 diabetes (T2D) and Cardiovascular diseases (CVD). Insulin resistance (IR) is frequently identified as a leading factor in these pathologies [1]. Use of proteomic and bioinformatic tools in protein expression studies enables greater understanding of biological mechanisms of complex diseases and also mechanisms of action of drugs and/or nutrients [2,3]. Proteins are the final and active product of most of the genome and thus, their levels are the most accurate reflection of what is happening when gene expression is modulated. Poor correlation between mRNA and protein expression, attributed to impaired translation efficiency [4], emphasizes the significance of directly determining protein abundance. Western blotting has been an effective tool for the study of protein expression for the last 30 years; however, it is limited by the size of the candidate pool that can be examined, giving an incomplete picture of the molecular phenotype.

In previous proteomic analyses, changes in the hepatic proteome in MetS, induced by high fat and high fructose diets in rodents [5,6], demonstrated modulation of proteins involved in glucose metabolism, lipid metabolism, oxidative stress and endoplasmic reticulum stress.

The feeding of polyphenol-rich plants, including those high in a subclass described as anthocyanins, has been shown to modify the protein and/or mRNA expression of several genes known to be involved in the processes of lipid metabolism, inflammation and energy homeostasis in the liver and/or adipose tissues [7,8,9,10,11,12,13]. These changes were associated with an improvement in various metabolic risk factors including glucose tolerance, insulin sensitivity, hyperlipidemia, hyperinsulinemia and hepatic steatosis [7,8,9,10,11,12,13]. However, to our knowledge, there has yet to be a study that examined whole proteomic changes in response to anthocyanin-rich plant-supplemented diets. Such a study would provide an unbiased and comprehensive picture of the molecular mechanisms responsible for these plants’ biological activity.

We previously showed that the substitution of the majority of carbohydrate in a high fat diet, with purple carrots (PC) or purple potatoes (PP), for 8 weeks, improved insulin sensitivity and blood pressure compared to a control high fat sucrose-rich diet in a model of MetS, obese Zucker rats. PP were more effective in improving insulin sensitivity while PC were more effective on the blood pressure measures [14]. The current study aimed to examine the proteomic changes in the liver and adipose tissues of these animals using tandem mass tag (TMT 10plex) labelling combined with liquid chromatography tandem mass spectrometry (LC-MS/MS). This technique enables the concurrent identification and comparative quantitation of the peptides from 10 different samples. These profiles are then used to generate potential molecular mechanisms for the observed phenotypic changes induced by these vegetables (i.e., improvement in insulin sensitivity and blood pressure).

## 2. Materials and Methods

### 2.1. Experimental Design, Sample Collection and Tissue Homogenization

Liver and adipose tissue samples were collected from rats *ad libitum* fed 3 exact experimental modified high fat AIN-93M diets (Research Diets Inc., New Brunswick, NJ, USA) (*n* = 15 rats/diet) that only differed for the carbohydrate source for 8 weeks (Table 1). The control diet had sucrose whereas PP and PC diets had purple potatoes and purple carrots as main sources of carbohydrate as previously described in detail [14]. This protocol was approved by the Animal Care Committee of the University of Guelph (Animal Utilization Protocol #12R012) in accordance with the guidelines from the Canadian Council on Animal Care (CCAC). A subsample of frozen liver (*n* = 3 per diet group) and adipose tissues (*n* = 3 per diet group) were randomly selected and homogenized (Fast Prep^®^ 24; MP biomedical, Santa Ana, CA, USA) using NP40 cell lysis buffer (Invitrogen, Camarillo, CA, USA) (3 volumes for adipose and 30 volumes for liver samples) supplemented with protease inhibitor cocktail and phenyl methyl sulfonyl fluoride (Sigma-Aldrich, St. Louis, MO, USA). The lysates were centrifuged at 5000× *g* for 10 min at 4 °C [15,16]. Total protein content of the infranatant was determined using a BCA protein assay kit (Thermo Fisher, Rockford, IL, USA). The lysates were then sent to the SPARC BioCentre, SickKids Hospital (Toronto, ON, Canada) to perform the TMT labelling and LC-MS/MS analyses.

### 2.2. Sample Preparation (Denaturation, Alkylation and Digestion) and TMT Labelling

The samples were solubilized with 1% Sodium dodecyl sulfate (SDS) and 8 M urea with sonication. The proteins were reduced in 1 mM dithiothreitol (DTT) for 1 h at 56 °C and the free cysteine residues were alkylated by incubating with iodoacetamide for 30 min protected from light at room temperature. The proteins were precipitated with 5 volumes of prechilled acetone overnight at −20 °C. The samples were centrifuged at 8000× *g* for 10 min at 4 °C. The pellets were dried for 2–3 min before dissolved with triethylammonium bicarbonate (TEAB). The samples were then digested with trypsin 2.5 µg for 100 µg of protein overnight at 37 °C. Fifty micrograms of protein from each sample was labeled using 0.4 mg of TMT 10plex (ThermoFisher, Rockford, IL, USA) by incubating at room temperature for 1 h. The labeling reaction was stopped using 5% hydroxylamine. The peptides were mixed and the solvent removed under vacuum.

### 2.3. Liquid Chromatography and Tandem Mass Spectrometry (LC-MS/MS)

The peptides were analyzed on an Orbitrap analyzer (Q-Exactive, ThermoFisher, San Jose, CA, USA) outfitted with a nanospray source and EASY-nLC nano-LC system (ThermoFisher, San Jose, CA, USA). a 75 μm × 50 cm PepMax RSLC EASY-Spray column filled with 2 μM C18 beads (ThermoFisher, SanJose, CA, USA) was used to load the peptide mixture at a pressure of 800 Bar. Peptides were then subjected to a stepwise gradient elution over 240 min at a rate of 250 nL/min (0–4% Acetonitrile containing 0.1% Formic Acid over 2 min; 4–28% Acetonitrile containing 0.1% Formic Acid over 226 min, 28–95% Acetonitrile containing 0.1% Formic Acid over 2 min, constant 95% Acetonitrile containing 0.1% Formic Acid for 10 min). In the Q-Exactive mass spectrometer (ThermoFisher, San Jose, CA, USA), one MS full scan (525–1600 m/z) was performed with an automatic gain control (AGC) target of 1 × 10^6^ maximum ion injection time of 120 ms and a resolution of 35,000 with subsequent 15 data-dependent MS/MS scans with a resolution of 35,000, an AGC target of 1 × 10^6^ , maximum ion time of 120 ms, and one microscan. The intensity threshold required to trigger a MS/MS scan was at an underfill ratio of 0.2%. In the higher energy collision dissociation (HCD) trap, normalized collision energy of 30 V was used for the fragmentation. The dynamic exclusion was applied with an exclusion period of 40 s [17].

### 2.4. Protein Identification and Quantitation

The MS/MS data was searched against the Rat UniProt database using Proteome Discoverer version 1.4 (ThermoFisher, San Jose, CA, USA) which also extracted the quantitation data from the 10 TMT tags. The data was imported into Scaffold Q+ (Proteome Software, Portland OR, USA) for label based quantitative analysis. Protein identifications were accepted if they contained at least 2 identified peptides above 95% tandem mass spectrometry confidence (with 0% decoy false discovery rate (FDR)). Differentially expressed proteins were determined by applying *t*-Test with unadjusted significance level *p* < 0.05 corrected by Benjamini–Hochberg.

### 2.5. In-Silico Functional Analyses

We performed in silico functional analyses of the differentially expressed proteins to explore the biological meaning behind the modulation of expression of these proteins by the purple vegetable diets. The Database for Annotation, Visualization and Integrated Discovery (DAVID) [18] was used to perform functional enrichment analyses. The enriched (i.e., overrepresented) Kyoto Encyclopedia of Genes and Genomes (KEGG) pathways and gene ontology (GO) terms biological processes component in the list of the differentially expressed proteins were identified. To account for multi-hypotheses testing, the *p*-values of the enrichment analyses were adjusted using Benjamini–Hochberg (*p* < 0.05).

## 3. Results and Discussion

### 3.1. Adipose Tissue Protein Expression

A total of 1944 proteins were identified in the adipose tissue of the rats fed the PP, the PC and the control diets (Supplemental Table S1), in which 85 and 224 proteins were differentially expressed with the PP and the PC diets respectively. 46 and 118 proteins were downregulated whereas 39 and 106 proteins were upregulated with the PP and the PC respectively (Table 2 and Table 3). 3 KEGG pathways and 220 biological processes were enriched in the proteins list of the PP diet while 24 KEGG pathways and 405 biological processes GO terms were enriched in the proteins list of the PC diet (at Benjamini *p* value < 0.05) (Supplemental Tables S2 and S5). Some of the enriched pathways and processes observed were mainly involved in lipid metabolism and cholesterol efflux with both diets and protein folding with the PP alone (Table 4 and Table 5).

#### 3.1.1. Protein Folding and Endoplasmic Reticulum (ER) Stress

“Protein processing in ER” pathway and “protein folding” biological process were both strongly enriched in the differentially expressed protein list with the PP (Table 4). All the proteins involved in both the pathway and the biological process were upregulated with the PP diet. UDP-glucose glycoprotein glucosyltransferase 1 (*Uggt1*), calnexin (*Canx*) and calreticulin (*Calr*) are involved in quality control process of protein folding in ER through recognizing, retaining and refolding the immaturely folded proteins [19]. *Uggt1* recognizes proteins with folding defects, retains them and directs them to *Canx*/*Calr* cycle to be refolded properly. Heat shock protein family A member 5 (*Hspa5*), PDI family (*Pdia 3*, *4 & 6*) and heat shock protein 90, beta, member 1 (*Hsp90b1*) are also recognized as major molecular chaperones [20]. Both *Hspa5* and *Hsp90b1* catalyze protein folding while the PDI family catalyzes the formation of disulphide bonds, thereby regulating regulates protein folding as well.

Accumulation of misfolded or unfolded proteins results in ER stress. ER stress response or UPR (unfolded protein response) is known as a common mechanism of the pathogenesis of IR. For instance, UPR recruits and activates a number of stress kinases that eventually impair insulin signaling pathway through inducing serine phosphorylation of IRS1. Moreover activation of the stress kinases promotes proinflammatory cytokines synthesis that also negatively affects insulin signaling [21]. Our finding is consistent with the observation that purple sweet potato color reduced the levels of the ER stress markers, phospho-pancreatic endoplasmic reticulum resident kinase (p-PERK), phospho-eukaryotic translation initiation factor (p-eIF2) and phopho-inositol-requiring 1 (p-IRE1) in the livers of mice fed high fat diet and also suppressed the ER induced inflammation by decreasing nuclear factor- κB (NF- κB) nuclear translocation [22].

#### 3.1.2. Lipid Metabolism

##### Lipid Synthesis

Both “fatty acid biosynthetic” and “lipid biosynthetic” processes were enriched in the differentially expressed protein list with both the PP and PC diets (Table 4 and Table 5). Among the proteins involved in these BP GO terms are Acetyl-CoA carboxylase alpha (*Acaca*) and fatty acid synthase (*Fasn*) that were downregulated with both diets. This indicated that de novo fatty acid synthesis was probably downregulated. ER lipid raft associated 2 (*Erlin2*) was upregulated with the PC diet. This can be another sign of a decreased de novo fatty acid synthesis with the PC diet. *Erlin2* depletion was shown to activate SREBP genes and subsequently increasing fatty acid and cholesterol biosynthesis [23]. However, the upregulation of phosphoenolpyruvate carboxykinase 1 (*Pck1*), with both diets, could be an indication of an increased fatty acid re-esterification, that could be coupled with the increased glyceroneogenesis. In adipose tissue, cytosolic *Pck1* is a key enzyme in glycerneogenesis that involves the synthesis of glycerol 3 phosphate (G-3-P) by decraboxylating amino acids to phosphoenolpyruvate (PEP) that then converts to dihydroxyacetone phosphate (DHAP), a precursor of G-3-p [24]. The synthesized G-3-P is utilized for fatty acid re-esterification and triglyceride (TG) synthesis in white adipose tissue [24]. In fact, over expression of *Pck1* in adipose tissue of mice was shown to increase FFA re-esterification, glycernoegenesis and obesity while decreasing circulating FFA levels and preserving glucose tolerance and whole body insulin sensitivity [25]. Lipid localization to the adipose tissue will probably decrease the lipid accumulation in other tissues (i.e., lipotoxicity). Intracellular accumulation of lipid intermediates like DAG and ceramides are known to interrupt insulin signaling [26]. Upregulation of Glycerol-3-phosphate acyltransferase 3 (*Gpat3*), with the PC diet, may be another sign of an increase in fatty acid re-esterification and TG synthesis. *Gpat3* is the first enzyme of the TG de novo synthesis pathway. Its increased expression increases TG formation [27]. Both apolipoprotein C1 (*Apoc1*) and apolipoprotein C2 (*Apoc2*) were upregulated with the PP diet whereas *Apoc2* and *Apoc3* were upregulated with the PC diet. *Apoc2* is required for lipoprotein lipase (LPL) activation. The LPL hydrolyzes TG to free fatty acids that are uptaken and deposited to the adipose tissue [28]. However, *Apoc1* and *Apoc3* exert the opposite effect of A*poc2* on LPL activity [29]. So it is not clear if LPL is activated or inhibited. Hydroxy-3-methylglutaryl-CoA synthase 2 (*Hmgcs2*) downregulation is an indication of a probable decrease in ketogenesis with both diets. *Hmgcs2* is a rate limiting enzyme of the ketone bodies biosynthesis [30]. It is a mitochondrial form of the enzyme that catalyzes the condensation of acetyl CoA with acetoacetyl CoA to form HMGCOA [30]. Ketogenesis is induced in long fasting, prolonged exercise and diabetes. Ketone bodies are used as fuels in these cases [30]. Also *Hmgcs2* expression increased with starvation and decreased in response to insulin [30]. So it seems that PP fed rats did not need ketone bodies for energy compared to the control group. Or perhaps they just had less acetyl CoA generated from β-oxidation that led to less ketone bodies synthesis.

##### Lipid Catabolism

The “lipid catabolic” process was enriched with both the PP and the PC while the “fatty acid catabolic” process was enriched with the PC alone and both “TG catabolic” and “TG metabolic” processes were enriched with the PP alone (Table 4 and Table 5). Upregulation of both perilipin1 (*Plin1*) and carboxylesterase 1D (*Ces1d*), with both diets, could be indicative of higher lipolysis activity. Both *Plin1* and *Ces1d* are known to be lipolytic proteins. However, *Plin1* has a complex role in lipolysis as it exerts opposing effects on basal and catecholamine stimulated lipolysis. Under basal state, *Plin1* decreases lipolysis through coating the lipid droplets and preventing the access of the lipolytic enzymes (e.g., hormone sensitive lipase) to the stored lipids. At the same time, TG levels are relatively unchanged. Most of the liberated FFAs, resulting from TG hydrolysis, are actually being recycled to TG [31] whereas, under stimulated conditions (i.e., during fasting or exercise), the phosphorylated *Plin1* gives access to hormone sensitive lipase and TG lipase to the lipid core allowing lipolysis [31]. Perillpin ablation in mice resulted in higher basal lipolysis and lower stimulated lipolysis. Perillipin null mice were lean but less glucose tolerant [32]. *Ces1d* was identified as a major lipolytic enzyme in mice [33]. However, it was not confirmed that it has the same effect on the lipolytic activity in human adipose tissue [34]. Furthermore, the concomitant upregulation of *Apoc2* and *Pck1* may support the idea that the liberated FFAs are not released to the circulation and instead they may actually be re-esterified and deposited to the adipose tissue. So generally we can see some evidence of lower FFA release and lower de novo fatty acid synthesis that may explain the improved insulin sensitivity with these diets.

Among the proteins involved in the “fatty acid catabolic” process are Acetyl-CoA acyltransferase 2 (*Acaa2*) and trifunctional protein (*Hadha & Hadhb*) and they were downregulated with the PC (Table 5). They are the enzymes catalyzing the last steps of the mitochondrial fatty acid β-oxidation. Peroxisomal bifunctional protein (*Ehhadh*) is also downregulated. *Ehhadh* is involved in peroxisomal fatty acid β-oxidation as well [35]. The probable decrease in the fatty acid oxidation observed may be due to either the reduced abundance of the newly synthesized fatty acids or directing fatty acids to the re-esterification pathway.

“Regulation of lipolysis in adipocytes” KEGG pathway is also enriched with the PC alone with GNAS complex locus (*Gnas*), abhydrolase domain containing 5 (*Abhd5*), hormone sensitive lipase (*Lipe*), cAMP-activated protein kinase (*Prkaca*) and *Plin1* (Table 5). They were all upregulated with the PC diet. This can be an indication of increased stimulated lipolysis in this group. Under catecholamine stimulation and during fasting, *Gnas* activates adenylate cyclase with a subsequent increase in cAMP. High levels of cAMP activate *Prkaca* that phosphorylates both *Lipe* and *Plin1* with a subsequent TG hydrolysis [36]. *Plin1* phosphorylation induces a conformational change that gives lipolytic enzymes more access to the adipocytes allowing lipolysis [37]. *Abhd5* also positively regulate lipolysis via activating adipose triglyceride lipase (ATGL). ATGL hydrolyzes TG releasing FFAs and DAG [38]. However, also only under the stimulated lipolysis state and *Plin1* phosphorylation, *Abhd5* gets released from its binding with *Plin1* which allows its action on ATGL [31]. During fasting or exercise, the liberated free fatty acids are needed and directed to other tissues to be oxidized for energy. Also *Plin1* upregulation may indicate less basal lipolysis. Higher basal lipolysis is suggested to be the cause of IR in *Plin1* null mice with low stimulated lipolysis [32]. However, more studies on differentiating the role of stimulated lipolysis versus the role of basal lipolysis in IR are needed.

#### 3.1.3. Cholesterol Efflux/Reverse Cholesterol Transport (RCT)

Both “cholesterol efflux” and “RCT” processes are enriched in the differentially expressed proteins list with the PP (Table 4). “Cholesterol efflux” process is also enriched with the PC (Table 5). Apolipoprotein A1 (*Apoa1*), *Apoa2*, *Apoc1* and *Apoc2* were upregulated while apolipoprotein E (*Apoe*) was downregulated with the PP diet. *Apoa2*, *Apoa4*, *Apoc2*, *Apoc3* and caveolin1 (*Cav1*) are all upregulated with the PC diet as well. Since *Apoa1* and *Apoa2* are the most abundant apolipoproteins in high density lipoprotein cholesterol containing particles (HDLc) [28], perhaps the higher protein abundance is simply an indication of overall higher HDLc levels with the PP compared to the control diet. As reported previously, the PP group was more insulin sensitive than the control group; it would not be surprising to see an associated improved lipid profile (i.e., higher HDLc). The association of dyslipidemia with IR is thought to be due to the high VLDL hepatic secretion and the high postprandial chylomicron levels coupled with the exchange of cholesterol esters from HDLc with TG from TG-rich lipoproteins. This leaves a more hydrolysis and dissociation prone TG-rich HDL particle, and thus reduces the number of HDL particles [39]. *Apoa1* transcription was shown to be modulated by dietary and hormonal factors [40]. Increased human *Apoa1* expression in transgenic mice increases HDLc levels and inhibits atherosclerosis [40]. At this point, it is not clear if the high *Apoa1* and *Apoa2* are the result of higher insulin sensitivity and higher HDLc with the PP diet, or due to a direct effect of the PP on the expression of *Apoa1* and *Apoa2*. Furthermore, since *Apoe* is typically found on TG-rich lipoproteins (chylomicrons, IDL, VLDL) [28], its decreased expression may be just a reflection of lower levels of these lipoproteins with the PP diet.

*Apoa1* has a major role in cholesterol efflux (i.e., cholesterol acceptor) and is also a main lecithin cholesterol acyl transferase (LACT) activator that catalyzes cholesterol esterification and promotes more cholesterol uptake by HDL particles [28]. However, *Apoa2*, *Apoa4*, *Apoc2*, *Apoc3* and *Cav1* were all shown to promote cholesterol efflux in vitro [41,42]. This strongly suggests that cholesterol efflux is enhanced with both diets. Cholesterol efflux is the first step of RCT that involves the removal of the excess cholesterol from the tissues and delivering it back to the liver for excretion [28].

Cholesterol efflux capacity was progressively reduced in patients with MetS with increasing number of MetS risk factors [43]. It also was negatively correlated with fasting blood glucose and systolic blood pressure [43]. Efflux capacity is inversely associated with the risk of coronary heart disease (CHD) [44]. Although the capacity is positively correlated with the Apoa1 concentration, it is the capacity, rather than the concentration, that is suggested to be the accurate predictor of CHD [44].

**Figure 1:**
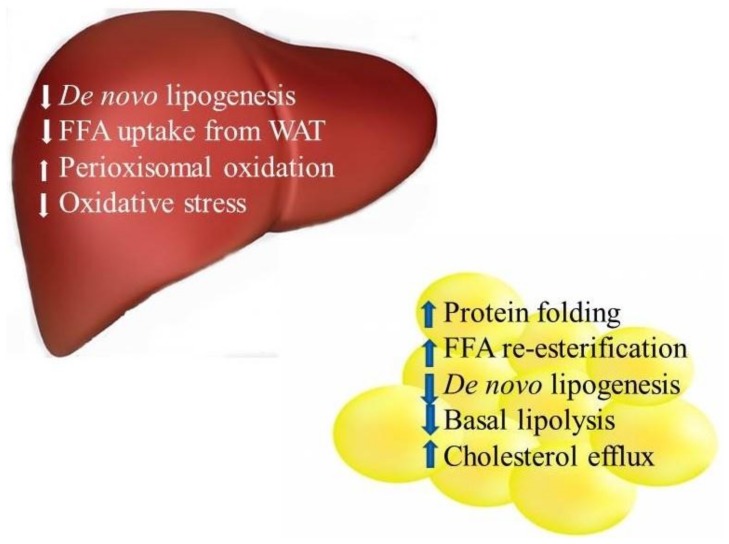
Suggested mechanisms of action of purple potatoes and purple carrots on Metabolic Syndrome pathologies in liver and adipose tissue. FFA: free fatty acids, WAT: white adipose tissue.

Taken together, these data suggest that decreased de novo lipogenesis, a decrease in basal lipolysis, increased fatty acid re-esterification, reduced ER stress (with PP alone), and probably increased cholesterol efflux in adipose tissue, each contributes to the mechanisms responsible for improving MetS pathologies (insulin sensitivity and hypertension), with PP and PC feeding (Figure 1).

### 3.2. Liver Protein Expression

A total of 941 proteins were identified in the livers of rats fed the PP, the PC and the control diets (Supplemental Table S3) of which 69 and 62 proteins were differentially expressed with the PP and the PC respectively. Thirty-seven proteins were downregulated and 32 proteins were upregulated with the PP diet (Table 6) whereas 29 proteins were downregulated and 33 proteins were upregulated with the PC diet (Table 7). A total of 26 KEGG pathways and 134 biological processes were enriched in the proteins list with the PP diet while 20 KEGG pathways and 130 biological processes were enriched with the PC diet (at Benjamini *p* value < 0.05) (Supplemental Tables S4 and S6). Some of the enriched pathways and processes observed were involved in lipid metabolism, carbohydrate metabolism and oxidative stress (Table 8 and Table 9).

#### 3.2.1. Lipid Metabolism

##### Lipid Synthesis

Both “fatty acid biosynthetic” and “lipid biosynthetic” processes are enriched in the list of the differentially expressed proteins with the PP while “acyl CoA biosynthetic” process was enriched with the PC diet (Table 8 and Table 9). Downregulation of *Fasn*, pyruvate carboxylase (*Pc*) and ATP citrate lyase (*Acly*) with the PP as well as downregulation of *Acly*, *Fasn* and pyruvate dehydrogenase alpha 1 (*Pdha1*) with the PC likely indicate a decrease in de novo fatty acid synthesis with both diets. *Pc* catalyzes the conversion of pyruvate to oxaloacetate that condenses with acetyl CoA to produce citrate. In the cytoplasm, *Acly* converts citrate back to acetyl CoA which is then used in fatty acid synthesis [45]. In db/db mice, ablation of hepatic citrate lyase prevents de novo lipogeneis and hepatic steotosis and promotes insulin sensitivity in muscle [46]. *Pdha1*, like *Acyl*, is an acetyl CoA source.

Farnesyl diphosphate synthase (*Fdps*) and solute carrier family 27 member 5 (*Slc27a5*) were both upregulated with the PP (Table 8). *Fdps* catalyzes the formation of farnesyl pyrophosphate that constitutes a branching point of the isoprenoid pathway that yield both sterol and non-sterol metabolites [47]. *Slc27a5* is a bile acyl CoA synthase that is involved in bile acid conjugation and activation before excretion into the bile canaliculi [48]. So, even though the upregulation of *Fdps* can be a sign of increased de novo cholesterol synthesis, the upregulation of *slc27a5* suggests an increased incorporation of the synthesized cholesterol into bile acid biosynthesis with the PP. Bile acid formation from cholesterol is a main cholesterol excretion route [47]. Primary bile acid synthesis was also enriched with the PC (Table 9). However, even though *Hsd17b4* is upregulated, *Akr1d1* and sterol carrier protein 2 (*Scp2*) are downregulated. All three proteins are involved in bile acid biosynthesis [49,50,51]. So no conclusion on bile acid synthesis can be made with the PC.

Both acyl-CoA synthetase long-chain family member 1 (*Acsl1*) and acyl-CoA synthetase long-chain family member 5 (*Acsl5*) are upregulated with the PP. Long chain acyl CoA synthases are a group of enzymes that catalyze the formation of acyl CoAs that can then be directed to either lipid synthesis or oxidation [52]. *Acsl1* is suggested to be mainly involved in TG synthesis whereas *Acls5* is suggested to be involved in β-oxidation [52]. However, data from a loss of function in vitro study, observed a role for *Acsl5* in directing fatty acids to TG synthesis [53]. In another loss of function study, hepatic *Acsl1* was suggested to have a role in both βoxidation and TG synthesis [54]. Because both pathways may be activated, it would be important to know the relative activation of one pathway over the other (i.e., enzyme activities and/or metabolite levels) to determine whether there would be overall change.

##### Lipid Catabolism

“Lipid catabolic” and “fatty acid β-oxidation” processes were enriched in the list of the differentially expressed proteins extracted from the liver tissues of the PP group while the “Fatty acid catabolic” process was enriched with that of the PC group (Table 8 and Table 9). Fatty acid β-oxidation seems to be downregulated with both diets. Acyl-CoA dehydrogenase, long chain (*Acadl*) was found to be downregulated with the PP. Also *Acadl*, *Acaa2*, and carnitine palmitoyltransferase 1A (*Cpt1a*) were all downregulated with the PC. *Acadl* and *Acaa2* catalyze the first and the last steps of β-oxidation pathway respectively whereas *Cpt1a* is the enzyme that is responsible for transporting fatty acids to the mitochondria for oxidation [35]. The probable decrease in the fatty acid oxidation could be due to the observed decrease in the abundance of the fatty acids as a result of reduced de novo lipogenesis. However, the Upregulation of acyl-CoA oxidase 3 (*Acox3*) and cytosolic isocitrate dehydrogenase (*Idh1*), with the PP, as well as, the upregulation of d bifunctional protein (*Hsd17b4*), with the PC, is probably a sign of higher peroxisomal fatty acid β-oxidation in the liver. *Acox3* is a rate limiting enzyme in β-oxidation pathway of the peroxisome as it catalyzes the oxidation of methyl branched fatty acyl CoAs and to a lesser extent straight chain fatty acids [35]. Also, cytosolic *Idh1* was shown to be necessary for peroxisomal β-oxidation of unsaturated fatty acids in rat liver cells through provision of NADPH [55]. *Hsd17b4* is also involved in peroxisomal fatty acid β-oxidation [49].

#### 3.2.2. Carbohydrate Metabolism

The “carbohydrate catabolic” process and “pentose phosphate” KEGG pathway were enriched with the PP while “carbohydrate metabolic” process was enriched with the PC (Table 8 and Table 9). Glycolysis seems to be decreased with both diets as glucose-6-phosphate isomerase (*Gpi*), fructose-bisphosphate aldolase B (*Aldob*) and pyruvate kinase (*Pklr*), 3 enzymes of the glycolytic pathway [56], and dihydrolipoamide S-acetyltransferase (*Dlat*) are all downregulated with the PP diet while both *Pklr* and *Pdha1* are downregulated with the PC diet. *Dlat* is a component of pyruvate dehydrogenase complex that converts pyruvate to acetyl CoA that gets directed to the citric acid cycle or used for de novo lipogenesis.

While glycolysis seems to be decreased, glycogen synthesis pathway proteins (i.e., glycogen synthase) do not seem to be higher in PP livers compared to control liver. However, it does seem that glucose is being directed to the pentose phosphate pathway, as glucose 6 phosphate dehydrogenase (*G6pd*) is upregulated with the PP. It is true that transketolase (*Tkt*) is downregulated but it is more involved in the non-oxidative part of the pathway that produces more glycolytic intermediates. The main products of the pentose phosphate pathway are NADPH and ribose 5 phosphate. NADPH is known to be used in fatty acid and cholesterol biosynthesis and in the reduction of oxidized glutathione [57]. Reduced glutathione may confer antioxidant protective effects as it reduces oxidized glutathione peroxidase [58]. It is worth noting that Glutathione peroxidase (*Gpx1*), the enzyme that reduces H_2_O_2_ [58], is also among the upregulated proteins in the PP list.

On the PC side, upregulation of UDP-glucose pyrophosphorylase 2 (*Ugp2*) may be a probable indication of increased glycogen synthesis with the PC. *Ugp2* catalyzes the reversible synthesis of UDP glucose which is the immediate precursor of glycogen synthesis [59]. Sorbitol dehydrogenase (*Sord*) is also downregulated with the PC. *Sord* is the second enzyme of the polyol pathway where glucose is converted to sorbitol then fructose by the action of *Sord*. However, its catalytic action is suggested to contribute to oxidative stress by producing NADH that produces ROS by the action of NADH oxidase [60].

#### 3.2.3. Oxidative Stress

“Response to oxidative stress” and “hydrogen peroxide catabolic” biological processes (Table 9) are enriched with the PC alone. Upregulation of catalase (*Cat*), enzyme catalyzing the conversion of H_2_O_2_ to water and O_2_ [61], can be a sign of antioxidant protective effects. Also, downregulation of both hemoglobin subunit beta and hemoglobin alpha 1 (*Hbb* and *Hba1*) may be a sign of less oxidative stress with PC group. The expression of both proteins was higher in fatty liver disease that was suggested to be due to the associated higher oxidative stress [62]. Similarly, heat shock protein family A (*Hsp70*) and heat shock protein family D member 1 (*Hspd1*) expression and phosphorylation respectively were induced in response to oxidative stress [63,64]. Parkinsonism associated deglycase (*Park7*) is a redox sensitive protein that was shown to be upregulated in vitro under oxidative stress conditions [65]. So downregulation of *Hspa8*, *Hspd1* and *Park7* may also be a sign of less oxidative stress with PC. Oxidative stress is an established player in promoting IR [66] and hypertension [67]. In fact, oxidative stress may be one of the links between fat accumulation in the liver and IR [68]. Oxidative stress interrupts insulin signaling through activating stress kinases and serine-phosphorylating IRS1 [68]. Furthermore, ROS induces endothelial dysfunction as one way of developing hypertension [67]. Oxidative stress is seen as a common pathological mechanism between fatty liver and CVD [69].

These findings are in agreement with multiple studies that observed antioxidative damage properties of purple vegetables. For instance, consumption of purple potatoes significantly reduced the concentrations of 8-hydroxydeoxyguanosine, a marker of oxidative stress induced DNA damage in men [70]. Purple carrot juice also decreased plasma oxidative stress markers such as malondialdehyde levels [71]. In vitro purple vegetable extracts were able to increase the activity of several antioxidant enzymes such as CAT, GPx and superoxide dismutase [72].

Taken together, these data suggest that a decrease in hepatic de novo lipogenesis, a probable increase in the peroxisomal fatty acid oxidation and a decrease in the fatty acid delivery to the liver from the adipose tissue, each contributes to the mechanisms responsible for improving MetS pathologies with PP and PC feeding (Figure 1). All of the aforementioned signs are mechanisms involved in hepatic lipid accumulation [73]. A decrease in hepatic de novo lipogenesis improves hepatic insulin sensitivity [73]. Lipid metabolites, such as DAG, induce IR in the liver by activating protein kinase C and serine-phosphorylating IRS1 [73]. Reducing oxidative damage may also be contributing to the positive effects of these vegetables on MetS pathologies in the liver (Figure 1).

Some of the current study findings are consistent with other proteomic studies that looked at the adipose proteomic profile changes in response to rosiglitazone [74], resveratrol [75], and caloric restriction [76]. The modulated proteins were involved in lipid metabolism such as perillpin with rosiglitazone [74] and APOA1, fatty acid binding proteins and aldoketoreductases with caloric restriction [76] and oxidative stress such as catalase and superoxide mutase with rosiglitazone [74] and perioxiredoxin and heat shock protein 70 with resveratrol [75]. Heat shock proteins involved in protein folding were also modulated with rosiglitazone [74].

## 4. Conclusions

There are some obvious similarities between the two purple vegetables in the enriched biological processes, the involved proteins and finally in the main suggested mechanisms of action in the liver and adipose tissue. Overall, we provided a molecular basis of the metabolic benefits of these vegetables that substantiate the results of our previous study on the metabolic phenotypic parameters. Interestingly, there appear to be many more regulated target proteins in the adipose tissue compared to the liver. This is somewhat surprising given the assumed central role for liver in handling macronutrients and phytochemicals. It does however, point to the now very much appreciated role of adipose tissue in regulating metabolism. No longer do we consider adipose as a benign fat depot but rather a pivotal regulator of the entire metabolic phenotype.

## Figures and Tables

**Table 1:** Composition of the experimental diets.

Component in g/kg Diet	Control	PP ^1^	PC ^2^
Casein (protein)	140	140	140
L-Cystine	1.8	1.8	1.8
Lard	120	120	120
Soybean Oil	40	40	40
Maltodextrin 10	150	150	150
Sucrose	450	-	150
Freeze dried baked purple potato	-	450	-
Freeze dried raw purple carrot	-	-	300
Cellulose, BW200	50	50	50
Vitamin Mix v10037	10	10	10
Mineral Mix s10022M	35	35	35
Choline bitartrate	2.5	2.5	2.5

^1^ PP is high fat diet supplemented with purple potatoes; ^2^ PC is high fat diet supplemented with purple carrots.

**Table 2:** Differentially expressed proteins with Purple Potatoes diet in adipose tissue.

Differentially Expressed Proteins	Gene Name	Log2 Fold Change ^1^	Up- or Down-Regulated	*p* Value ^2^
Serum albumin precursor	*Alb*	−0.17	down	0.0001
Serotransferrin precursor	*Tf*	−0.33	down	0.0001
Fatty acid synthase	*Fasn*	−0.26	down	0.0001
Myosin-9	*Myh9*	−0.14	down	0.0001
Alpha-1-macroglobulin precursor	*A1m*	0.32	up	0.0001
Fibrillin-1 isoform X1	*Fbn1*	−0.12	down	0.0001
Filamin-A isoform X2	*Flna*	−0.06	down	0.0001
Spectrin beta chain, non-erythrocytic 1 isoform X1	*SPTBN1*	0.07	up	0.0001
78 kDa glucose-regulated protein precursor	*Hspa5*	0.3	up	0.0001
Membrane primary amine oxidase	*Aoc3*	0.11	up	0.0001
Calreticulin precursor	*Calr*	0.33	up	0.0001
Transketolase isoform X1	*Tkt*	−0.19	down	0.0001
Endoplasmin precursor	*Hsp90b1*	0.33	up	0.0001
Inter-alpha-trypsin inhibitor heavy chain H4 precursor	*Itih4*	0.12	up	0.0001
Carboxylesterase 1D precursor	*Ces1d*	0.38	up	0.0001
Pyruvate kinase PKM isoform X2	*Pkm*	−0.11	down	0.0001
Apolipoprotein A-I preproprotein	*Apoa1*	0.26	up	0.0001
Hemopexin precursor	*Hpx*	−0.3	down	0.0001
Cofilin-1	*Cfl1*	−0.16	down	0.0001
Vitamin D-binding protein precursor	*Gc*	−0.14	down	0.0001
Fibrinogen beta chain precursor	*Fgb*	0.2	up	0.0001
Myoferlin	*Myof*	−0.18	down	0.0001
Hypoxia up-regulated protein 1 isoform X1	*Hyou1*	0.38	up	0.0001
Plastin-3 isoform X2	*Pls3*	0.29	up	0.0001
Complement factor B precursor	*Cfb*	−0.25	down	0.0001
Carbamoyl-phosphate synthase [ammonia], mitochondrial precursor	*Cps1*	−0.38	down	0.0001
Fibrinogen gamma chain isoform X1	*Fgg*	0.22	up	0.0001
Dolichyl-diphosphooligosaccharide-protein glycosyltransferase subunit 2 isoform X1	*Rpn2*	0.26	up	0.0001
UDP-glucose:glycoprotein glucosyltransferase 1 precursor	*Uggt1*	0.15	up	0.0001
Protein disulfide-isomerase A6 precursor	*Pdia6*	0.31	up	0.0001
Dolichyl-diphosphooligosaccharide-protein glycosyltransferase subunit 1 precursor	*Rpn1*	0.22	up	0.0001
Adipocyte plasma membrane-associated protein isoform X2	*Apmap*	0.16	up	0.0001
Acetyl-coa carboxylase 1	*Acaca*	−0.26	down	0.0001
Apolipoprotein E precursor	*Apoe*	−0.4	down	0.0001
Fibrinogen alpha chain isoform 2 precursor	*Fga*	0.21	up	0.0001
Catechol O-methyltransferase isoform X1	*Comt*	−0.21	down	0.0001
Peroxiredoxin-5, mitochondrial precursor	*Prdx5*	−0.29	down	0.0001
Phosphoenolpyruvate carboxykinase, cytosolic [GTP]	*Pck1*	0.19	up	0.0001
Coronin-1A isoform X1	*CORO1A*	−0.24	down	0.0001
Hydroxymethylglutaryl-coa synthase, mitochondrial isoform X1	*Hmgcs2*	−0.25	down	0.0001
Complement component C7 isoform X1	*C7*	−0.25	down	0.0001
Perilipin-2	*Plin2*	−0.4	down	0.0001
Galectin-3	*Lgals3*	−0.44	down	0.0001
Integrin alpha-M isoform X1	*Itgam*	−0.3	down	0.0001
Brain acid soluble protein 1	*Basp1*	0.19	up	0.0001
Carbonyl reductase [NADPH] 1	*LOC102556347*	0.27	up	0.0001
Apolipoprotein C-II precursor	*Apoc2*	0.46	up	0.0001
Laminin subunit alpha-4 precursor	*Lama4*	0.1	up	0.0002
Protein disulfide-isomerase A3 precursor	*Pdia3*	0.22	up	0.0002
Cathepsin D precursor	*Ctsd*	−0.23	down	0.0002
Macrophage mannose receptor 1 precursor	*Mrc1*	−0.14	down	0.0003
Filamin-B	*Flnb*	0.11	up	0.0003
3-ketoacyl-coa thiolase, mitochondrial	*Acaa2*	−0.14	down	0.0003
Chloride intracellular channel protein 1	*Clic1*	−0.15	down	0.0003
Integrin beta-2 precursor	*Itgb2*	−0.26	down	0.0003
Cystatin-B	*Cstb*	−0.23	down	0.0003
Von Willebrand factor A domain-containing protein 5A isoform X2	*LOC108348048*	−0.16	down	0.0003
Apolipoprotein A-II isoform X1	*Apoa2*	0.41	up	0.0003
Neutral alpha-glucosidase AB isoform X1	*Ganab*	0.14	up	0.0004
Transaldolase	*Taldo1*	−0.14	down	0.0004
Tissue alpha-L-fucosidase precursor	*Fuca1*	0.76	up	0.0004
Phosphatidylethanolamine-binding protein 1	*Pebp1*	0.36	up	0.0004
Apolipoprotein C-I precursor	*Apoc1*	0.39	up	0.0005
Protein disulfide-isomerase A4 precursor	*Pdia4*	0.29	up	0.0006
Selenium-binding protein 1 isoform X1	*LOC103689947*	−0.12	down	0.0006
Heat shock 70 kDa protein 1A	*Hspa1b*	0.12	up	0.0006
Ester hydrolase c11orf54 homolog	*RGD1309534*	−0.15	down	0.0006
Complement C3 precursor	*C3*	−0.15	down	0.0007
Reticulocalbin-1 precursor	*Rcn1*	0.29	up	0.0007
Histidine-trna ligase, cytoplasmic	*Hars*	0.27	up	0.0007
Transmembrane glycoprotein NMB precursor	*Gpnmb*	−0.3	down	0.0009
Rho GDP-dissociation inhibitor 2 isoform X1	*Arhgdib*	−0.22	down	0.0010
Granulins isoform a precursor	*Grn*	−0.23	down	0.0011
Betaine-homocysteine S-methyltransferase 1	*Bhmt*	−0.32	down	0.0011
Plastin-2 isoform X2	*Lcp1*	−0.14	down	0.0012
Transgelin-2 isoform X1	*Tagln2*	−0.15	down	0.0012
Calnexin isoform X1	*Canx*	0.13	up	0.0013
Nucleolin	*Ncl*	−0.13	down	0.0016
Prothymosin alpha	*Ptma*	−0.14	down	0.0016
ATP synthase subunit d, mitochondrial	*Atp5h*	−0.11	down	0.0017
Alpha-1-acid glycoprotein precursor	*Orm1*	−0.36	down	0.0017
Perilipin-1 isoform X1	*Plin1*	0.1	up	0.0018
NAD(P)H-hydrate epimerase	*Naxe*	0.18	up	0.0018
Fructose-bisphosphate aldolase A isoform X2	*Aldoa*	−0.09	down	0.0019
Cysteine sulfinic acid decarboxylase isoform X1	*Csad*	0.12	up	0.0019

^1^ Log2 Fold Change by Category (Purple Potatoes/Control); ^2^ *p* value of the *t*-test less than 5% Benjamini–Hochberg threshold (0.0022).

**Table 3:** Differentially expressed proteins with the Purple Carrots Diet in adipose tissue.

Defferntially Expressed Proteins	Gene Name	Log2 Fold Change ^1^	Down- or Up-Regulated	*p* Value ^2^
Serum albumin precursor	*Alb*	−0.19	down	0.0001
Serotransferrin precursor	*Tf*	−0.22	down	0.0001
Fatty acid synthase	*Fasn*	−0.15	down	0.0001
Myosin-9	*Myh9l1*	−0.07	down	0.0001
Elongation factor 1-alpha 1	*Eef1a1*	−0.11	down	0.0001
Filamin-A isoform X2	*Flna*	−0.09	down	0.0001
Alpha-enolase	*Eno1*	−0.15	down	0.0001
Ribosome-binding protein 1 isoform X4	*Rrbp1*	−0.16	down	0.0001
Plastin-2 isoform X2	*Lcp1*	−0.17	down	0.0001
Aldehyde dehydrogenase, mitochondrial precursor	*Aldh2*	−0.16	down	0.0001
Collagen alpha-1 (XIV) chain precursor	*Col14a1*	−0.44	down	0.0001
ATP-citrate synthase isoform X1	*Acly*	−0.26	down	0.0001
Glutamate dehydrogenase 1, mitochondrial precursor	*Mrc1*	−0.14	down	0.0001
Carbamoyl-phosphate synthase [ammonia], mitochondrial precursor	*Cps1*	−0.75	down	0.0001
Heterogeneous nuclear ribonucleoprotein U	*Hnrnpu*	−0.2	down	0.0001
Serine protease inhibitor A3N	*Serpina3n*	−0.27	down	0.0001
Decorin isoform X1	*Dcn*	−0.4	down	0.0001
Glutathione S-transferase alpha-3	*Gsta1*	−0.27	down	0.0001
Prolargin isoform X3	*Prelp*	−0.29	down	0.0001
3-ketoacyl-coa thiolase, mitochondrial	*Acaa2*	−0.29	down	0.0001
Acetyl-coa carboxylase 1	*Acaca*	−0.18	down	0.0001
Aspartate aminotransferase, mitochondrial	*Got2*	−0.21	down	0.0001
Heterogeneous nuclear ribonucleoprotein K isoform X2	*Hnrnpk*	−0.18	down	0.0001
ATP synthase subunit d, mitochondrial	*Atp5h*	−0.14	down	0.0001
Catechol O-methyltransferase isoform X1	*Comt*	−0.34	down	0.0001
Nucleolin	*Ncl*	−0.32	down	0.0001
Hydroxymethylglutaryl-coa synthase, mitochondrial isoform X1	*Hmgcs2*	−0.47	down	0.0001
Complement component C7 isoform X1	*C7*	−0.21	down	0.0001
Galectin-3	*Lgals3*	−0.34	down	0.0001
Biglycan precursor	*Bgn*	−0.24	down	0.0001
Granulins isoform a precursor	*Grn*	−0.33	down	0.0001
Ezrin	*Ezr*	−0.24	down	0.0001
Nucleophosmin	*Npm1*	−0.33	down	0.0001
Elongation factor Tu, mitochondrial precursor	*Tufm*	−0.12	down	0.0001
Beta-2-glycoprotein 1 precursor	*Apoh*	−0.37	down	0.0001
Betaine-homocysteine S-methyltransferase 1	*Bhmt*	−0.69	down	0.0001
Obg-like atpase 1	*Ola1*	−0.14	down	0.0001
Glutathione S-transferase Mu 1	*Gstm1*	−0.62	down	0.0001
High mobility group box 1 like	*Hmg1l1*	−0.4	down	0.0001
Alcohol dehydrogenase 1	*Adh1*	−0.75	down	0.0001
Fatty acid-binding protein, liver	*Fabp1*	−0.77	down	0.0001
Von Willebrand factor A domain-containing protein 5A isoform X2	*LOC108348048*	−0.17	down	0.0001
Serine/threonine-protein kinase N3	*Pkn3*	−0.26	down	0.0001
Heterogeneous nuclear ribonucleoprotein M isoform b	*Hnrnpm*	−0.22	down	0.0001
Argininosuccinate synthase isoform X1	*Ass1*	−0.53	down	0.0001
Fructose-bisphosphate aldolase B	*Aldob*	−0.65	down	0.0001
LIM and senescent cell antigen-like-containing domain protein 1	*Lims1*	−0.17	down	0.0001
Arginase-1	*Arg1*	−0.5	down	0.0001
Sorbitol dehydrogenase	*Sord*	−0.31	down	0.0001
Carbonic anhydrase 3 isoform X1	*Car3*	0.15	up	0.0001
Vimentin	*Vim*	0.23	up	0.0001
Long-chain-fatty-acid-coa ligase 1 isoform X1	*Acsl1*	0.12	up	0.0001
Alpha-1-macroglobulin precursor	*Pzp*	0.21	up	0.0001
Fibrillin-1 isoform X1	*Fbn1*	0.15	up	0.0001
Complement C3 precursor	*C3*	0.14	up	0.0001
Spectrin beta chain, non-erythrocytic 1 isoform X1	*Sptbn1*	0.1	up	0.0001
Plectin isoform 1	*Plec*	0.06	up	0.0001
Membrane primary amine oxidase	*Aoc3*	0.21	up	0.0001
All-trans-retinol 13,14-reductase precursor	*Retsat*	0.17	up	0.0001
Collagen alpha-3(VI) chain isoform X4	*Col6a3*	0.12	up	0.0001
Vinculin	*Vcl*	0.12	up	0.0001
Carboxylesterase 1D precursor	*Ces1d*	0.32	up	0.0001
Perilipin-1 isoform X1	*Plin1*	0.19	up	0.0001
Complement C4 precursor	*C4a*	0.25	up	0.0001
Malate dehydrogenase, cytoplasmic isoform Mdh1	*Mdh1*	0.12	up	0.0001
EH domain-containing protein 1	*Ehd1*	0.13	up	0.0001
Catalase	*Cat*	0.14	up	0.0001
Laminin subunit alpha-4 precursor	*Lama4*	0.27	up	0.0001
Laminin subunit beta-1 isoform X2	*Lamb1*	0.26	up	0.0001
Laminin subunit gamma-1 precursor	*Lamc1*	0.21	up	0.0001
Aldose reductase	*Akr1b1*	0.17	up	0.0001
Periostin isoform X2	*Postn*	0.25	up	0.0001
Hormone-sensitive lipase	*Lipe*	0.18	up	0.0001
L-lactate dehydrogenase B chain isoform Ldhb	*Ldhb*	0.15	up	0.0001
Succinyl-coa:3-ketoacid coenzyme A transferase 1, mitochondrial precursor	*Oxct1*	0.15	up	0.0001
Dolichyl-diphosphooligosaccharide-protein glycosyltransferase subunit 2 isoform X1	*Rpn2*	0.12	up	0.0001
Cysteine sulfinic acid decarboxylase isoform X1	*Csad*	0.16	up	0.0001
Cell surface glycoprotein MUC18 isoform 1 precursor	*Mcam*	0.24	up	0.0001
Adipocyte plasma membrane-associated protein isoform X2	*Apmap*	0.19	up	0.0001
Alanine aminotransferase 1 isoform X1	*Gpt*	0.19	up	0.0001
Nidogen-1 isoform X2	*Nid1*	0.18	up	0.0001
Fibrinogen alpha chain isoform 2 precursor	*Fga*	0.17	up	0.0001
Annexin A3 isoform X1	*Anxa3*	0.16	up	0.0001
Glutathione peroxidase 3 precursor	*Gpx3*	0.2	up	0.0001
Phosphoenolpyruvate carboxykinase, cytosolic [GTP]	*Pck1*	0.32	up	0.0001
Perilipin-4 isoform X2	*Plin4*	0.21	up	0.0001
Laminin subunit alpha-2 isoform X1	*Lama2*	0.23	up	0.0001
Heat shock protein beta-1	*Hspb1*	0.27	up	0.0001
Integrin alpha-7 isoform X1	*Itga7*	0.18	up	0.0001
Acetolactate synthase-like protein	*Ilvbl*	0.21	up	0.0001
Caveolin-1 isoform alpha	*Cav1*	0.29	up	0.0001
Ras-related protein Rab-18 isoform X1	*Rab18*	0.16	up	0.0001
Apolipoprotein A-II isoform X1	*Apoa2*	0.37	up	0.0001
1-acyl-sn-glycerol-3-phosphate acyltransferase gamma	*Agpat3*	0.18	up	0.0001
GNAS isoform GNASL	*Gnas*	0.22	up	0.0001
Chloride intracellular channel protein 1	*Clic1*	−0.15	down	0.0001
Neprilysin isoform X1	*Mme*	0.24	up	0.0001
Creatine kinase B-type	*Ckb*	−0.15	down	0.0001
Protein S100-B isoform X1	*S100b*	0.17	up	0.0001
Fibrinogen beta chain precursor	*Fgb*	0.14	up	0.0001
Calumenin isoform a precursor	*Calu*	0.15	up	0.0001
T-complex protein 1 subunit zeta	*Cct6a*	−0.12	down	0.0001
Hepatoma-derived growth factor	*Hdgf*	−0.3	down	0.0001
Transaldolase	*Taldo1*	−0.13	down	0.0002
Sorbin and SH3 domain-containing protein 2	*Sorbs2*	0.29	up	0.0002
Fibrinogen gamma chain isoform X1	*Fgg*	0.14	up	0.0002
Dysferlin	*Dysf*	0.16	up	0.0002
Aminoacyl trna synthase complex-interacting multifunctional protein 1	*Aimp1*	−0.32	down	0.0002
Apolipoprotein C-III precursor	*Apoc3*	0.25	up	0.0002
Heat shock 70 kDa protein 1A	*Hspa1b*	0.11	up	0.0002
Transmembrane protein 43	*Tmem43*	0.14	up	0.0002
Monoglyceride lipase isoform X1	*Mgll*	0.14	up	0.0002
Apolipoprotein A-IV precursor	*Apoa4*	0.15	up	0.0002
Alcohol dehydrogenase [NADP(+)]	*Akr1a1*	−0.13	down	0.0002
Glucose-6-phosphate isomerase	*Gpi*	0.13	up	0.0002
Lumican precursor	*Lum*	−0.18	down	0.0003
Glutamine synthetase	*Glul*	−0.22	down	0.0003
PDZ and LIM domain protein 1	*Pdlim1*	0.25	up	0.0003
Filamin-B	*Flnb*	0.1	up	0.0003
Legumain precursor	*Lgmn*	−0.17	down	0.0003
RNA-binding protein FUS isoform X1	*Fus*	−0.2	down	0.0003
Septin-9 isoform 2	*Sept9*	−0.17	down	0.0003
Delta-1-pyrroline-5-carboxylate dehydrogenase, mitochondrial	*Aldh4a1*	−0.32	down	0.0003
Cadherin-13 precursor	*Cdh13*	0.25	up	0.0003
Apolipoprotein C-II precursor	*Apoc2*	0.34	up	0.0003
Protein-glutamine gamma-glutamyltransferase 2	*Tgm2*	−0.3	down	0.0003
Glutathione S-transferase Mu 2	*Gstm2*	−0.25	down	0.0004
60S ribosomal protein L5	*Rpl5*	−0.18	down	0.0004
Transketolase isoform X1	*Tkt*	−0.1	down	0.0005
Synapse-associated protein 1 isoform X1	*Syap1*	0.2	up	0.0005
Sulfated glycoprotein 1 isoform X1	*Psap*	−0.32	down	0.0005
Camp-dependent protein kinase type II-beta regulatory subunit	*Prkar2b*	0.15	up	0.0005
Proliferation-associated protein 2G4	*Pa2g4*	−0.27	down	0.0005
L-lactate dehydrogenase A chain isoform X1	*Ldha*	−0.14	down	0.0005
Unconventional myosin-Ic	*Myo1c*	0.07	up	0.0006
Prelamin-A/C	*Lmna*	0.1	up	0.0006
Phosphoserine aminotransferase	*Psat1*	0.15	up	0.0006
Isocitrate dehydrogenase [NADP], mitochondrial precursor	*Idh2*	−0.23	down	0.0006
Reticulon-4	*Rtn4*	0.18	up	0.0006
Transmembrane glycoprotein NMB precursor	*Gpnmb*	−0.27	down	0.0006
Nucleobindin-1 isoform X1	*Nucb1*	0.13	up	0.0006
Retinol dehydrogenase 11 precursor	*Rdh11*	0.28	up	0.0006
Poly [ADP-ribose] polymerase 3	*Parp3*	−0.19	down	0.0007
Hsc70-interacting protein	*St13*	0.11	up	0.0007
40S ribosomal protein S19	*Rps19*	−0.23	down	0.0007
Alpha-actinin-4	*Actn4*	0.08	up	0.0007
Serine hydroxymethyltransferase, cytosolic	*Shmt1*	−0.25	down	0.0008
Cofilin-1	*Cfl1*	−0.12	down	0.0009
Lamin-B1	*Lmnb1*	0.17	up	0.0010
Heterogeneous nuclear ribonucleoprotein A3 isoform a	*Hnrnpa3*	−0.26	down	0.0010
Polymerase I and transcript release factor	*Ptrf*	0.12	up	0.0010
Ras gtpase-activating-like protein IQGAP1	*Iqgap1*	−0.07	down	0.0011
Probable ATP-dependent RNA helicase DDX5 isoform X1	*Ddx5*	−0.14	down	0.0011
Eukaryotic initiation factor 4A-II isoform X1	*Eif4a2*	0.12	up	0.0011
Moesin	*Msn*	−0.14	down	0.0012
Ribonuclease UK114	*Rida*	−0.32	down	0.0012
Dynactin subunit 2	*Dctn2*	0.1	up	0.0012
Splicing factor U2AF 65 kDa subunit isoform X1	*U2af2*	−0.18	down	0.0013
Annexin A1 isoform X2	*Anxa1*	0.11	up	0.0013
ATP synthase subunit O, mitochondrial precursor	*Atp5o*	−0.13	down	0.0014
Uncharacterized protein LOC315963	*RGD1310507*	−0.14	down	0.0014
Coagulation factor XIII A chain	*F13a1*	0.16	up	0.0014
1-acylglycerol-3-phosphate O-acyltransferase ABHD5	*Abhd5*	0.16	up	0.0014
Receptor of activated protein C kinase 1	*Rack1*	−0.16	down	0.0015
Ethylmalonyl-coa decarboxylase isoform X2	*Echdc1*	0.15	up	0.0015
Peptidyl-prolyl cis-trans isomerase FKBP9 precursor	*Fkbp9*	0.2	up	0.0015
Glutathione S-transferase Mu 5	*Got2*	−0.43	down	0.0016
ATP synthase-coupling factor 6, mitochondrial isoform X2	*Atp5j*	−0.13	down	0.0016
Epididymal secretory protein E1 precursor	*Npc2*	−0.15	down	0.0016
Glycerol-3-phosphate acyltransferase 3 isoform X1	*Gpat3*	0.27	up	0.0016
60S ribosomal protein L4	*Rpl4*	−0.13	down	0.0017
Carbonyl reductase [NADPH] 1	*LOC102556347*	0.24	up	0.0017
Transmembrane protein 120A	*Tmem120a*	0.33	up	0.0017
Annexin A5	*Anxa5*	0.12	up	0.0019
Trifunctional enzyme subunit alpha, mitochondrial precursor	*Hadha*	−0.08	down	0.0021
Sorbin and SH3 domain-containing protein 1 isoform X6	*Sorbs1*	0.16	up	0.0021
Long-chain fatty acid transport protein 3 precursor	*Slc27a3*	0.22	up	0.0021
Ceruloplasmin isoform 1 precursor	*Cp*	−0.08	down	0.0022
Heterogeneous nuclear ribonucleoproteins C1/C2-like isoform X5	*LOC100911576*	−0.13	down	0.0022
Peroxisomal bifunctional enzyme	*Ehhadh*	−0.29	down	0.0022
Fructose-1,6-bisphosphatase 1	*Fbp1*	−0.52	down	0.0024
Aconitate hydratase, mitochondrial precursor	*Aco2*	0.08	up	0.0025
General vesicular transport factor p115 isoform X1	*Uso1*	0.14	up	0.0025
Antigen-presenting glycoprotein CD1d precursor	*Cd1d1*	0.18	up	0.0025
Bifunctional glutamate/proline-trna ligase isoform X1	*Eprs*	−0.12	down	0.0027
Alpha-2-HS-glycoprotein precursor	*Ahsg*	−0.27	down	0.0027
Macrophage mannose receptor 1 precursor	*Mrc1*	−0.09	down	0.0028
Peptidyl-prolyl cis-trans isomerase B precursor	*Ppib*	−0.17	down	0.0028
40S ribosomal protein S9	*LOC103689992*	−0.13	down	0.0028
Aldehyde dehydrogenase family 8 member A1	*Aldh8a1*	−0.8	down	0.0028
Erlin-2 isoform X1	*Erlin2*	0.1	up	0.0028
Peroxiredoxin-5, mitochondrial precursor	*Prdx5*	−0.18	down	0.0029
Pantetheinase precursor	*Vnn1*	0.24	up	0.0029
Adenosylhomocysteinase	*Ahcy*	−0.18	down	0.0030
3-oxo-5-beta-steroid 4-dehydrogenase	*Akr1d1*	−0.44	down	0.0030
Septin-11	*Sept11*	−0.14	down	0.0032
Cathepsin D precursor	*Ctsd*	−0.16	down	0.0033
ATP synthase subunit delta, mitochondrial isoform X1	*Atp5d*	0.1	up	0.0033
Coronin-1A isoform X1	*Coro1A*	−0.14	down	0.0034
Calcium-binding mitochondrial carrier protein Aralar2 isoform X1	*Slc25a13*	−0.33	down	0.0034
Annexin A6	*Anxa6*	0.08	up	0.0034
40S ribosomal protein S15	*Rps15*	0.23	up	0.0034
Mitochondrial dicarboxylate carrier	*Slc25a10*	−0.12	down	0.0035
Serum deprivation-response protein	*Sdpr*	0.12	up	0.0035
Ras-related protein Rab-2A	*Rab2a*	0.12	up	0.0035
Platelet endothelial cell adhesion molecule precursor	*Pecam1*	0.17	up	0.0036
Glyceraldehyde-3-phosphate dehydrogenase	*Gapdh*	−0.09	down	0.0038
Peptidyl-prolyl cis-trans isomerase A	*LOC100360977*	−0.14	down	0.0039
Actin-related protein 2/3 complex subunit 1B	*Arpc1b*	−0.17	down	0.0040
Thiosulfate sulfurtransferase	*Tst*	−0.2	down	0.0040
Guanine nucleotide-binding protein G(I)/G(S)/G(T) subunit beta-1	*Gnb1*	0.14	up	0.0040
Phenylalanine-4-hydroxylase	*Pah*	−0.4	down	0.0046
Talin-1	*Tln1*	−0.05	down	0.0048
60S ribosomal protein L30	*Rpl30*	−0.14	down	0.0048
Erythrocyte band 7 integral membrane protein	*Stom*	0.26	up	0.0048
Camp-dependent protein kinase catalytic subunit alpha	*Prkaca*	0.23	up	0.0050
Calcineurin B homologous protein 1	*Chp1*	0.15	up	0.0050
Trifunctional enzyme subunit beta, mitochondrial isoform X2	*Hadhb*	−0.1	down	0.0052
Transmembrane 9 superfamily member 3 isoform X2	*Tm9sf3*	−0.22	down	0.0052
Peroxiredoxin-1	*Prdx1*	−0.12	down	0.0053
UDP-glucuronosyltransferase 2B2 precursor	*Ugt2b*	−0.64	down	0.0053
Carbonyl reductase [NADPH] 3	*Cbr3*	0.14	up	0.0055
Guanylate-binding protein 4 isoform X1	*LOC685067*	0.15	up	0.0056
Creatine kinase M-type	*Ckm*	0.19	up	0.0057

^1^ Log2 Fold Change by Category (Purple Carrots/Control); ^2^ *p* value of the *t*-test less than 5% Benjamini–Hochberg threshold (0.0058).

**Table 4:** Enriched gene ontology biological process terms and KEGG pathways in the list of differentially expressed proteins with Purple Potatoes in adipose tissue that are involved in protein folding, lipid metabolism and cholesterol efflux.

Biological Theme	GO (BP) and KEGG Pathway ^1^	Gene Names ^2^	*p*-Value ^3^
Protein Folding	GO:0006457~protein folding	***Uggt1***, ***Canx***, ***Calr***, ***Hspa1b***, ***Hsp90b1***, ***Pdia3***, ***Pdia4***, ***Pdia6***	1.46 × 10^−6^
	rno04141~Protein processing in endoplasmic reticulum	***Uggt1***, ***Canx***, ***Calr***, ***Ganab***, ***Hspa1b***, ***Hsp90b1***, ***Hspa5***, ***Hyou1***, ***Pdia3***, ***Pdia4***, ***Pdia6***, ***Rpn1***, ***Rpn2***	7.66 × 10^−10^
Lipid Metabolism	GO:0006633~fatty acid biosynthetic process	*Acaca*, ***Apoa1***, ***Apoc1***, ***Apoc2***, *Fasn*	2.95 × 10^−3^
	GO:0008610~lipid biosynthetic process	*Hmgcs2*, *Acaca*, ***Apoa1***, ***Apoc1***, ***Apoc2***, *Apoe*, *C3*, *Fasn*, ***Pck1***	2.29 × 10^−3^
	GO:0016042~lipid catabolic process	***Apoa1***, ***Apoa2***, ***Apoc1***, *Apoe*, *Cps1*, ***Ces1d***, ***Plin1***	2.71 × 10^−4^
	GO:0006641~triglyceride metabolic process	***Apoa1***, ***Apoc1***, ***Apoc2***, *Apoe*, *Cps1*, *C3*, ***Plin1***, ***Pck1***	2.65 × 10^−7^
Cholesterol efflux	GO:0033344~cholesterol efflux	***Apoa1***, ***Apoa2***, ***Apoc1***, ***Apoc2***, *Apoe*	3.68 × 10^−5^
	GO:0043691~reverse cholesterol transport	***Apoa1***, ***Apoa2***, *Apoe*	1.34 × 10^−3^

^1^ GO (BP) is Gene Ontology (GO) biological process component (BP) and KEGG pathway is Kyoto Encyclopedia of Genes and Genomes biological pathway; ^2^ Gene names in bold are upregulated with Purple Potatoes diet while the un-bold names are downregulated with the Purple Potatoes diet in adipose tissue; ^3^ *p*-value of the enrichment analyses is significant at Benjamini <0.05.

**Table 5:** Enriched gene ontology biological process terms and KEGG pathways in the list of differentially expressed proteins with Purple Carrots in adipose tissue that are involved in lipid metabolism and cholesterol efflux.

Biological Theme	GO (BP) and KEGG Pathway ^1^	Gene Names ^2^	*p*-Value ^3^
Lipid Metabolism	GO:0006633~fatty acid biosynthetic process	***Erlin2***, *Acaca*, ***Anxa1***, ***Apoa4***, ***Apoc2***, ***Apoc3***, *Fasn*, ***Mgll***	9.44 × 10^−4^
	GO:0008610~lipid biosynthetic process	*Hmgcs2*, ***Erlin2***, ***Abhd5***, *Acaca*, ***Acsl1***, *Aldh8a1*, *Akr1d1*, ***Anxa1***, ***Apoa4***, ***Apoc2***, ***Apoc3***, ***C3***, *Fasn*, ***Gpat3***, ***Mgll***, ***Pck1***	1.14 × 10^−3^
	GO:0016042~lipid catabolic process	***Abhd5***, *Acaa2*, *Akr1d1*, ***Apoa2***, ***Apoa4***, ***Apoc2***, ***Apoc3***, *Apoh*, *Cps1*, ***Ces1d***, *Ehhadh*, *Fabp1*, *Hadha*, *Hadhb*, ***Lipe***, ***Mgll***, ***Plin1***, ***Prkaca***	3.35 × 10^−8^
	GO:0009062~fatty acid catabolic process	*Acaa2*, ***Ces1d***, *Ehhadh*, *Fabp1*, *Hadha*, *Hadhb*, ***Lipe***	5.28 × 10^−4^
	rno04923:Regulation of lipolysis in adipocytes	***Gnas***, ***Abhd5***, ***Lipe***, ***Mgll***, ***Plin1***, ***Prkaca***	5.01 × 10^−3^
Cholesterol efflux	GO:0033344~cholesterol efflux	*Npc2*, ***Apoa2***, ***Apoa4***, ***Apoc2***, ***Apoc3***, ***Cav1***	1.20 × 10^−4^

^1^ GO (BP) is Gene Ontology (GO) biological process component (BP) and KEGG pathway is Kyoto Encyclopedia of Genes and Genomes biological pathway; ^2^ Gene names in bold are upregulated with the Purple Carrots diet while the un-bold names are downregulated with the Purple Carrots diet in adipose tissue; ^3^ *p*-value of the enrichment analyses is significant at Benjamini <0.05.

**Table 6:** Differentially expressed proteins with Purple Potatoes diet in liver.

Differentially Expressed Proteins	Gene Name	Log2 Fold Change ^1^	Up- or Down-Regulated	*p* Value ^2^
Carbamoyl-phosphate synthase [ammonia], mitochondrial	*Cps1*	0.17	up	0.0001
Fatty acid-binding protein, liver	*Fabp1*	0.27	up	0.0001
Long-chain-fatty-acid-CoA ligase 1	*Acsl1*	0.1	up	0.0001
Bucs1 protein	*Acsm1*	0.19	up	0.0001
3-alpha-hydroxysteroid dehydrogenase	*Akr1c9*	0.17	up	0.0001
Aldh4a1 protein (Fragment)	*Aldh4a1*	0.13	up	0.0001
Alpha-aminoadipic semialdehyde dehydrogenase	*Aldh7a1*	0.17	up	0.0001
Cystathionine gamma-lyase	*Cth*	0.2	up	0.0001
Microsomal triglyceride transfer protein	*Mttp*	0.15	up	0.0001
Long-chain-fatty-acid-CoA ligase 5	*Acsl5*	0.24	up	0.0001
Bile acyl-CoA synthetase	*Slc27a5*	0.22	up	0.0001
Alcohol sulfotransferase A	*St2a2*	0.43	up	0.0001
Aldose reductase-related protein 1	*Akr1b7*	1.41	up	0.0001
Fatty acid synthase	*Fasn*	−0.18	down	0.0001
Pyruvate carboxylase, mitochondrial	*Pc*	−0.09	down	0.0001
Serum albumin	*Alb*	−0.13	down	0.0001
Triokinase/FMN cyclase	*Tkfc*	−0.14	down	0.0001
Transketolase	*Tkt*	−0.13	down	0.0001
ATP-citrate synthase	*Acly*	−0.27	down	0.0001
Serotransferrin	*Tf*	−0.24	down	0.0001
Pyruvate kinase	*Pklr*	−0.18	down	0.0001
Selenium-binding protein 1	*Selenbp1*	−0.14	down	0.0001
Glucose-6-phosphate isomerase	*Gpi*	−0.18	down	0.0001
Purine nucleoside phosphorylase	*Pnp*	−0.12	down	0.0001
Malate dehydrogenase, mitochondrial	*Mdh2*	−0.22	down	0.0001
Keratin, type II cytoskeletal 8	*Krt8*	−0.2	down	0.0001
Glycerol kinase	*Gk*	−0.16	down	0.0001
Cytochrome P450 2C11	*Cyp2c11*	−0.37	down	0.0001
Keratin, type I cytoskeletal 18	*Krt18*	−0.23	down	0.0001
Phosphate carrier protein, mitochondrial	*Slc25a3*	−0.2	down	0.0001
Isoform 2 of Fibrinogen beta chain	*Fgb*	0.27	up	0.0001
Acyl-coenzyme A synthetase ACSM5, mitochondrial	*Acsm5*	−0.35	down	0.0001
Farnesyl pyrophosphate synthase 1	*Fdps*	0.21	up	0.0001
Protein disulfide-isomerase	*P4hb*	0.1	up	0.0002
Choline dehydrogenase, mitochondrial	*Chdh*	−0.13	down	0.0002
Carboxylesterase 1D	*Ces1d*	0.36	up	0.0002
Malate dehydrogenase, cytoplasmic	*Mdh1*	0.15	up	0.0003
Malic enzyme	*Me1*	−0.15	down	0.0003
Glutathione peroxidase	*Gpx1*	0.17	up	0.0003
Aflatoxin B1 aldehyde reductase member 3	*Akr7a3*	−0.26	down	0.0004
Lactamase, beta	*Lactb*	−0.14	down	0.0004
Alpha-aminoadipic semialdehyde synthase, mitochondrial	*Aass*	0.22	up	0.0005
Perilipin 2	*Plin2*	−0.41	down	0.0005
Acyl-coenzyme A oxidase	*Acox3*	0.09	up	0.0005
Kynurenine/alpha-aminoadipate aminotransferase, mitochondrial	*Aadat*	0.18	up	0.0005
Dihydrolipoyllysine-residue acetyltransferase component of pyruvate dehydrogenase complex, mitochondrial	*Dlat*	−0.17	down	0.0006
Carboxylic ester hydrolase (Fragment)	*Ces2e*	0.49	up	0.0009
Cytochrome P450 2B3	*Cyp2b3*	0.18	up	0.0009
Estrogen sulfotransferase, isoform 3	*Ste*	−0.46	down	0.001
Glucose-6-phosphate 1-dehydrogenase	*G6pdx*	0.35	up	0.001
Alcohol dehydrogenase 1	*Adh1*	0.08	up	0.0012
Isocitrate dehydrogenase [NADP] cytoplasmic	*Idh1*	0.09	up	0.0012
Glutathione S-transferase alpha-4	*Gsta4*	0.13	up	0.0012
Myosin, heavy polypeptide 9, non-muscle	*Myh9*	−0.1	down	0.0012
Protein deglycase DJ-1	*Park7*	−0.26	down	0.0012
Transgelin-2	*Tagln2*	−0.21	down	0.0013
Phosphoenolpyruvate carboxykinase, cytosolic [GTP]	*Pck1*	0.11	up	0.0014
Long-chain specific acyl-CoA dehydrogenase, mitochondrial	*Acadl*	−0.1	down	0.0014
Voltage-dependent anion-selective channel protein 3	*Vdac3*	−0.27	down	0.0017
Alpha-1-macroglobulin	*A1m*	0.14	up	0.0018
Aflatoxin B1 aldehyde reductase member 2	*Akr7a2*	−0.15	down	0.0019
Fructose-bisphosphate aldolase	*Aldob*	−0.11	down	0.0021
Epoxide hydrolase 1	*Ephx1*	−0.11	down	0.0021
UDP-glucuronosyltransferase 2B2	*Ugt2b*	0.17	up	0.0023
3 beta-hydroxysteroid dehydrogenase type 5	*Hsd3b5*	−0.24	down	0.0024
3-hydroxyisobutyryl-CoA hydrolase, mitochondrial	*Hibch*	−0.16	down	0.0027
Cytosol aminopeptidase	*Lap3*	−0.08	down	0.0028
UDP-glucuronosyltransferase 2B17 OS	*Ugt2b17*	0.27	up	0.003
Biliverdin reductase A	*Blvra*	−0.15	down	0.0033

^1^ Log2 Fold Change by Category (Purple Potatoes/Control); ^2^ *p* value of the *t*-test less than 5% Benjamini–Hochberg threshold (0.0037).

**Table 7:** Differentially expressed proteins with the Purple Carrots diet in liver.

Differentially Expressed Proteins	Gene Name	Log2 Fold Change ^1^	Up- or Down-Regulated	*p* Value ^2^
Carbamoyl-phosphate synthase [ammonia], mitochondrial	*Cps1*	0.05	up	0.0001
Cytosolic 10-formyltetrahydrofolate dehydrogenase	*Aldh1l1*	0.14	up	0.0001
Catalase	*Cat*	0.15	up	0.0001
Cytochrome P450 2C7	*Cyp2c7*	0.29	up	0.0001
Alcohol dehydrogenase 1	*Adh1*	0.14	up	0.0001
Alpha-1-macroglobulin	*A1m*	0.14	up	0.0001
Epoxide hydrolase 1	*Ephx1*	0.21	up	0.0001
Cystathionine gamma-lyase	*Cth*	0.19	up	0.0001
4-hydroxyphenylpyruvate dioxygenase	*Hpd*	0.25	up	0.0001
Glutathione S-transferase	*Gsta5*	0.46	up	0.0001
Protein Sar1a	*Sar1a*	0.18	up	0.0001
Aflatoxin B1 aldehyde reductase member 3	*Akr7a3*	0.45	up	0.0001
Histidine ammonia-lyase	*Hal*	0.35	up	0.0001
Carboxylesterase 1D	*Ces1d*	0.54	up	0.0001
Fatty acid synthase	*Fasn*	−0.13	down	0.0001
Aldehyde dehydrogenase, mitochondrial	*Aldh2*	−0.25	down	0.0001
3-ketoacyl-CoA thiolase, mitochondrial	*Acaa2*	−0.27	down	0.0001
60 kDa heat shock protein, mitochondrial	*Hspd1*	−0.07	down	0.0001
Transketolase	*Tkt*	−0.25	down	0.0001
ATP-citrate synthase	*Acly*	−0.3	down	0.0001
Malate dehydrogenase, mitochondrial	*Mdh2*	−0.22	down	0.0001
Keratin, type II cytoskeletal 8	*Krt8*	−0.13	down	0.0001
Sorbitol dehydrogenase	*Sord*	−0.14	down	0.0001
Aldehyde dehydrogenase X, mitochondrial	*Aldh1b1*	−0.46	down	0.0001
Protein LOC679794	*LOC679794*	−0.33	down	0.0001
UDP-glucuronosyltransferase 2B2	*Ugt2b*	0.18	up	0.0002
Hemoglobin subunit beta-1	*Hbb*	−0.24	down	0.0002
Pyruvate kinase	*Pklr*	−0.12	down	0.0002
Protein Ugp2	*Ugp2*	0.25	up	0.0002
Isoform 2 of Fibrinogen beta chain	*Fgb*	0.21	up	0.0002
UDP-glucuronosyltransferase 2B15	*Ugt2b15*	0.13	up	0.0003
Alpha-aminoadipic semialdehyde synthase, mitochondrial	*Aass*	0.17	up	0.0004
Cytochrome P450 2C23	*Cyp2c23*	0.2	up	0.0004
Argininosuccinate synthase	*Ass1*	0.11	up	0.0004
Pyruvate dehydrogenase E1 component subunit alpha	*Pdha1*	−0.22	down	0.0004
Keratin, type I cytoskeletal 18	*Krt18*	−0.17	down	0.0006
3-oxo-5-beta-steroid 4-dehydrogenase	*Akr1d1*	−0.08	down	0.0006
3-alpha-hydroxysteroid dehydrogenase	*Akr1c9*	0.1	up	0.0007
Perilipin 2	*Plin2*	−0.31	down	0.0007
Hemoglobin subunit alpha-1/2	*Hba1*	−0.22	down	0.0007
Long-chain specific acyl-CoA dehydrogenase, mitochondrial	*Acadl*	−0.1	down	0.0008
Carnitine O-palmitoyltransferase 1, liver isoform	*Cpt1a*	−0.22	down	0.0009
L-gulonolactone oxidase	*Gulo*	−0.17	down	0.0009
Retinol dehydrogenase 7	*Rdh7*	0.15	up	0.0010
Protein deglycase DJ-1	*Park7*	−0.17	down	0.0010
Peroxisomal multifunctional enzyme type 2	*Hsd17b4*	0.07	up	0.0015
60S ribosomal protein L14	*Rpl14*	−0.17	down	0.0015
Glutathione S-transferase	*Gsta2*	0.53	up	0.0017
Malate dehydrogenase, cytoplasmic	*Mdh1*	0.13	up	0.0017
Probable 2-oxoglutarate dehydrogenase E1 component DHKTD1, mitochondrial	*Dhtkd1*	0.12	up	0.0017
3-hydroxy-3-methylglutaryl-Coenzyme A synthase 2 (Mitochondrial)	*Hmgcs2*	−0.07	down	0.0017
Pterin-4-alpha-carbinolamine dehydratase	*Pcbd1*	−0.2	down	0.0017
Heat shock cognate 71 kDa protein	*Hspa8*	−0.06	down	0.0018
Non-specific lipid-transfer protein	*Scp2*	−0.15	down	0.0020
Carbonic anhydrase 3	*Ca3*	0.48	up	0.0022
Protein LOC100911833	*LOC297568*	0.14	up	0.0023
Cytochrome P450 2A2	*Cyp2a2*	0.38	up	0.0023
Cullin-associated NEDD8-dissociated protein 1	*Cand1*	−0.2	down	0.0023
Eukaryotic translation elongation factor 1 beta 2	*Eef1b2*	−0.16	down	0.0023
Ectonucleoside triphosphate diphosphohydrolase 5	*Entpd5*	0.17	up	0.0027
Glutathione S-transferase alpha-5	*Gsta5*	0.33	up	0.0027
Formimidoyltransferase-cyclodeaminase	*Ftcd*	0.06	up	0.0033

^1^ Log2 Fold Change by Category (Purple Carrots/Control); ^2^ *p* value of the *t*-test less than 5% Benjamini–Hochberg threshold (0.00336).

**Table 8:** Enriched gene ontology biological process terms and KEGG pathways in the list of differentially expressed proteins with Purple Potatoes in liver that are involved in lipid metabolism and carbohydrate metabolism.

Biological Them	GO (BP) and KEGG Pathway ^1^	Gene Names ^2^	*p*-Value ^3^
Lipid Metabolism	GO:0006633~fatty acid biosynthetic process	*Acly*, *Acadl*, ***Acsm1***, *Acsm5*, *Fasn*	1.58× 10^−3^
	GO:0008610~lipid biosynthetic process	*Hsd3b5*, *Acly*, *Acadl*, ***Acsl1***, ***Acsl5***, ***Acsm1***, *Acsm5*, ***Fdps***, *Fasn*, ***G6pd***, ***Idh1***, ***Pck1***, *Pc*, ***Slc27a5***	6.00 × 10^−8^
	GO:0016042~lipid catabolic process	*Hibch*, *Acadl*, ***Acox3***, ***Acsl5***, ***Cps1***, ***Ces1d***, ***Fabp1***, ***Idh1***	7.64 × 10^−5^
	GO:0006635~fatty acid beta-oxidation	*Hibch, Acadl, **Acox3, Acsl5, Ces1d, Fabp1***	1.32 × 10^−4^
Carbohydrate Metabolism	GO:0016052~carbohydrate catabolic process	*Aldob*, ***Cps1***, *Gpi*, *Gk*, *Pklr*	1.42 × 10^−3^
	rno00030:Pentose phosphate pathway	*Aldob*, ***G6pd***, *Gpi*, *Tkt*	1.19× 10^−3^

^1^ GO (BP) is Gene Ontology (GO) biological process component (BP) and KEGG pathway is Kyoto Encyclopedia of Genes and Genomes biological pathway; ^2^ Gene names in bold are upregulated with Purple Potatoes diet while the un-bold names are downregulated with the Purple Potatoes diet in liver; ^3^ *p*-value of the enrichment analyses is significant at Benjamini < 0.05.

**Table 9:** Enriched gene ontology biological process terms and KEGG pathways in the list of differentially expressed proteins with Purple Carrots in liver that are involved in lipid metabolism, carbohydrate metabolism and oxidative stress.

Biological Theme	GO (BP) and KEGG Pathway ^1^	Gene Names ^2^	*p*-Value ^3^
Lipid Metabolism	GO:0009062~fatty acid catabolic process	*Acaa2*, *Acadl*, ***Ces1d***, *Cpt1a*,***Hsd17b4***	2.46 × 10^−4^
	GO:0071616~acyl-CoA biosynthetic process	*Acly*, *Fasn*, *Pdha1*, *Pdha1l1*	1.85 × 10^−4^
	rno00120:Primary bile acid biosynthesis	*Akr1d1*, ***Hsd17b4***, *Scp2*	4.77 × 10^−3^
Carbohydrate Metabolism	GO:0005975~carbohydrate metabolic process	***Ugp2***, ***Cps1***, *Cpt1a*,***Dhtkd1***,***Entpd5***,***Mdh1,** Mdh2*, *Pdha1*, *Pklr*, *Sord*	8.83 × 10^−4^
Oxidative Stress	GO:0006979~response to oxidative stress	*Park7*, ***Car3***, ***Cat***, *Hsp70*, *Hspa8*, *Hspd1*, *Hbb*, *Hba1*	1.60 × 10^−3^
	GO:0042744~hydrogen peroxide catabolic process	***Cat***, *Hbb*, *Hba1*	2.55 × 10^−3^

^1^ GO (BP) is Gene Ontology (GO) biological process component (BP) and KEGG pathway is Kyoto Encyclopedia of Genes and Genomes biological pathway; ^2^ Gene names in bold are upregulated with the Purple Carrots diet while the un-bold names are downregulated with the Purple Carrots diet in liver; ^3^ *p*-value of the enrichment analyses is significant at Benjamini < 0.05.

## Supplementary Materials

The following are available online at http://www.mdpi.com/2072-6643/10/4/456/s1, Table S1: The list of identified proteins in the adipose tissue, Table S2: Enriched gene ontology biological process terms and pathways in the list of the differentially expressed proteins with purple potatoes diet in adipose tissue, Table S3: The list of identified proteins in liver, Table S4: Enriched gene ontology biological process terms in the list of the differentially expressed proteins with purple potatoes diet in liver, Table S5: Enriched gene ontology biological process terms and pathways in the list of the differentially expressed proteins with the purple carrots diet in adipose tissue, Table S6: Enriched gene ontology biological process terms in the list of the differentially expressed proteins with the purple carrots diet in liver.

## References

[1] Gallagher E.J., LeRoith D., Karnieli E. (2010). Insulin resistance in obesity as the underlying cause for the metabolic syndrome. Mt. Sinai J. Med. J. Transl. Pers. Med..

[2] Rubio-Aliaga I., Silva-Zolezzi I., Affolter M., Dayon L., Panchaud A., Kussmann M. (2014). Proteomics in the systems-level study of the metabolic syndrome. A Systems Biology Approach to Study Metabolic Syndrome.

[3] Kim E.Y., Kim W.K., Oh K.-J., Han B.S., Lee S.C., Bae K.-H. (2015). Recent advances in proteomic studies of adipose tissues and adipocytes. Int. J. Mol. Sci..

[4] Maier T., Güell M., Serrano L. (2009). Correlation of mRNA and protein in complex biological samples. FEBS Lett..

[5] Hsieh C.-C., Liao C.-C., Liao Y.-C., Hwang L.S., Wu L.-Y., Hsieh S.-C. (2016). Proteomic changes associated with metabolic syndrome in a fructose-fed rat model. J. Food Drug Anal..

[6] Luo M., Mengos A.E., Stubblefield T.M., Mandarino L.J. (2012). High fat diet-induced changes in hepatic protein abundance in mice. J. Proteom. Bioinform..

[7] Seymour E., Lewis S.K., Urcuyo-Llanes D.E., Tanone I.I., Kirakosyan A., Kaufman P.B., Bolling S.F. (2009). Regular tart cherry intake alters abdominal adiposity, adipose gene transcription, and inflammation in obesity-prone rats fed a high fat diet. J. Med. Food.

[8] Seymour E.M., Singer A.A., Kirakosyan A., Urcuyo-Llanes D.E., Kaufman P.B., Bolling S.F. (2008). Altered hyperlipidemia, hepatic steatosis, and hepatic peroxisome proliferator-activated receptors in rats with intake of tart cherry. J. Med. Food.

[9] Tsuda T., Horio F., Uchida K., Aoki H., Osawa T. (2003). Dietary cyanidin 3-O-β-D-glucoside-rich purple corn color prevents obesity and ameliorates hyperglycemia in mice. J. Nutr..

[10] Peng C.-H., Liu L.-K., Chuang C.-M., Chyau C.-C., Huang C.-N., Wang C.-J. (2011). Mulberry water extracts possess an anti-obesity effect and ability to inhibit hepatic lipogenesis and promote lipolysis. J. Agric. Food Chem..

[11] DeFuria J., Bennett G., Strissel K.J., Perfield J.W., Milbury P.E., Greenberg A.S., Obin M.S. (2009). Dietary blueberry attenuates whole-body insulin resistance in high fat-fed mice by reducing adipocyte death and its inflammatory sequelae. J. Nutr..

[12] Takikawa M., Inoue S., Horio F., Tsuda T. (2010). Dietary anthocyanin-rich bilberry extract ameliorates hyperglycemia and insulin sensitivity via activation of AMP-activated protein kinase in diabetic mice. J. Nutr..

[13] Tsuda T., Ueno Y., Aoki H., Koda T., Horio F., Takahashi N., Kawada T., Osawa T. (2004). Anthocyanin enhances adipocytokine secretion and adipocyte-specific gene expression in isolated rat adipocytes. Biochem. Biophys. Res. Commun..

[14] Ayoub H.M., McDonald M.R., Sullivan J.A., Tsao R., Platt M., Simpson J., Meckling K.A. (2017). The effect of anthocyanin-rich purple vegetable diets on metabolic syndrome in obese Zucker rats. J. Med. Food.

[15] MacPherson R., Huber J.S., Frendo-Cumbo S., Simpson J.A., Wright D.C. (2015). Adipose tissue insulin action and IL-6 signaling after exercise in obese mice. Med. Sci. Sports Exerc..

[16] Castellani L., Perry C.G., Macpherson R.E., Root-McCaig J., Huber J.S., Arkell A.M., Simpson J.A., Wright D.C. (2015). Exercise-mediated IL-6 signaling occurs independent of inflammation and is amplified by training in mouse adipose tissue. J. Appl. Physiol..

[17] Mahadevan V., Khademullah C.S., Dargaei Z., Chevrier J., Uvarov P., Kwan J., Bagshaw R.D., Pawson T., Emili A., De Koninck Y. (2017). Native KCC2 interactome reveals PACSIN1 as a critical regulator of synaptic inhibition. eLife.

[18] Huang D.W., Sherman B.T., Lempicki R.A. (2009). Systematic and integrative analysis of large gene lists using DAVID bioinformatics resources. Nat. Protoc..

[19] Malhotra J.D., Kaufman R.J. (2007). The endoplasmic reticulum and the unfolded protein response. Seminars in Cell & Developmental Biology.

[20] Naidoo N. (2009). ER and aging—Protein folding and the ER stress response. Ageing Res. Rev..

[21] Flamment M., Hajduch E., Ferré P., Foufelle F. (2012). New insights into ER stress-induced insulin resistance. Trends Endocrinol. Metab..

[22] Wang X., Zhang Z.-F., Zheng G.-H., Wang A.-M., Sun C.-H., Qin S.-P., Zhuang J., Lu J., Ma D.-F., Zheng Y.-L. (2017). The inhibitory effects of purple sweet potato color on hepatic inflammation is associated with restoration of NAD+ levels and attenuation of NLRP3 inflammasome activation in high-fat-diet-treated mice. Molecules.

[23] Huber M.D., Vesely P.W., Datta K., Gerace L. (2013). Erlins restrict SREBP activation in the ER and regulate cellular cholesterol homeostasis. J. Cell Biol..

[24] Nye C., Kim J., Kalhan S.C., Hanson R.W. (2008). Reassessing triglyceride synthesis in adipose tissue. Trends Endocrinol. Metab..

[25] Franckhauser S., Muñoz S., Pujol A., Casellas A., Riu E., Otaegui P., Su B., Bosch F. (2002). Increased fatty acid re-esterification by PEPCK overexpression in adipose tissue leads to obesity without insulin resistance. Diabetes.

[26] Turban S., Hajduch E. (2011). Protein kinase C isoforms: Mediators of reactive lipid metabolites in the development of insulin resistance. FEBS Lett..

[27] Cao J., Li J.-L., Li D., Tobin J.F., Gimeno R.E. (2006). Molecular identification of microsomal acyl-CoA: Glycerol-3-phosphate acyltransferase, a key enzyme in de novo triacylglycerol synthesis. Proc. Natl. Acad. Sci. USA.

[28] Tulenko T.N., Sumner A.E. (2002). The physiology of lipoproteins. J. Nucl. Cardiol..

[29] Larsson M., Vorrsjö E., Talmud P., Lookene A., Olivecrona G. (2013). Apolipoproteins CI and C-III inhibit lipoprotein lipase activity by displacement of the enzyme from lipid droplets. J. Biol. Chem..

[30] Hegardt F.G. (1999). Mitochondrial 3-hydroxy-3-methylglutaryl-CoA synthase: A control enzyme in ketogenesis. Biochem. J..

[31] MacPherson R.E., Peters S.J. (2015). Piecing together the puzzle of perilipin proteins and skeletal muscle lipolysis. Appl. Physiol. Nutr. Metab..

[32] Tansey J., Sztalryd C., Gruia-Gray J., Roush D., Zee J., Gavrilova O., Reitman M., Deng C.-X., Li C., Kimmel A. (2001). Perilipin ablation results in a lean mouse with aberrant adipocyte lipolysis, enhanced leptin production, and resistance to diet-induced obesity. Proc. Natl. Acad. Sci. USA.

[33] Soni K.G., Lehner R., Metalnikov P., O’Donnell P., Semache M., Gao W., Ashman K., Pshezhetsky A.V., Mitchell G.A. (2004). Carboxylesterase 3 (EC 3.1. 1.1) is a major adipocyte lipase. J. Biol. Chem..

[34] Jernås M., Olsson B., Arner P., Jacobson P., Sjöström L., Walley A., Froguel P., McTernan P.G., Hoffstedt J., Carlsson L.M. (2009). Regulation of carboxylesterase 1 (CES1) in human adipose tissue. Biochem. Biophys. Res. Commun..

[35] Reddy J.K., Hashimoto T. (2001). Peroxisomal *β*-oxidation and peroxisome proliferator–activated receptor α: An adaptive metabolic system. Annu. Rev. Nutr..

[36] Ahmadian M., Wang Y., Sul H.S. (2010). Lipolysis in adipocytes. Int. J. Biochem. Cell Biol..

[37] Braseamble D. (2007). The perilipin family of structural lípid droplet proteins: Stabilization of lípid droplets and control of lipolysis. J. Lipid Res..

[38] Lass A., Zimmermann R., Haemmerle G., Riederer M., Schoiswohl G., Schweiger M., Kienesberger P., Strauss J.G., Gorkiewicz G., Zechner R. (2006). Adipose triglyceride lipase-mediated lipolysis of cellular fat stores is activated by CGI-58 and defective in chanarin-dorfman syndrome. Cell Metab..

[39] Miccoli R., Bianchi C., Penno G., Del Prato S. (2008). Insulin resistance and lipid disorders. Future Lipidol..

[40] Lewis G.F., Rader D.J. (2005). New insights into the regulation of HDL metabolism and reverse cholesterol transport. Circ. Res..

[41] Lin Y.-C., Ma C., Hsu W.-C., Lo H.-F., Yang V.C. (2007). Molecular interaction between caveolin-1 and ABCA1 on high-density lipoprotein-mediated cholesterol efflux in aortic endothelial cells. Cardiovasc. Res..

[42] Remaley A.T., Stonik J.A., Demosky S.J., Neufeld E.B., Bocharov A.V., Vishnyakova T.G., Eggerman T.L., Patterson A.P., Duverger N.J., Santamarina-Fojo S. (2001). Apolipoprotein specificity for lipid efflux by the human ABCAI transporter. Biochem. Biophys. Res. Commun..

[43] Gall J., Frisdal E., Bittar R., Le Goff W., Bruckert E., Lesnik P., Guerin M., Giral P. (2016). Association of cholesterol efflux capacity with clinical features of metabolic syndrome: Relevance to atherosclerosis. J. Am. Heart Assoc..

[44] Saleheen D., Scott R., Javad S., Zhao W., Rodrigues A., Picataggi A., Lukmanova D., Mucksavage M.L., Luben R., Billheimer J. (2015). Association of HDL cholesterol efflux capacity with incident coronary heart disease events: A prospective case-control study. Lancet Diabetes Endocrinol..

[45] Bauer D.E., Hatzivassiliou G., Zhao F., Andreadis C., Thompson C.B. (2005). ATP citrate lyase is an important component of cell growth and transformation. Oncogene.

[46] Wang Q., Jiang L., Wang J., Li S., Yu Y., You J., Zeng R., Gao X., Rui L., Li W. (2009). Abrogation of hepatic ATP-citrate lyase protects against fatty liver and ameliorates hyperglycemia in leptin receptor-deficient mice. Hepatology.

[47] Russell D.W. (1992). Cholesterol biosynthesis and metabolism. Cardiovasc. Drugs Ther..

[48] Mihalik S.J., Steinberg S.J., Pei Z., Park J., Kim D.G., Heinzer A.K., Dacremont G., Wanders R.J., Cuebas D.A., Smith K.D. (2002). Participation of two members of the very long-chain acyl-CoA synthetase family in bile acid synthesis and recycling. J. Biol. Chem..

[49] Möller G., Van Grunsven E., Wanders R., Adamski J. (2001). Molecular basis of d-bifunctional protein deficiency. Mol. Cell. Endocrinol..

[50] Seltman H., Diven W., Rizk M., Noland B., Chanderbhan R., Scallen T., Vahouny G., Sanghvi A. (1985). Regulation of bile-acid synthesis. Role of sterol carrier protein2 in the biosynthesis of 7α-hydroxycholesterol. Biochem. J..

[51] Kondo K.H., Kai M.H., Setoguchi Y., Eggertsen G., Sjöblom P., Setoguchi T., Okuda K.I., Björkhem I. (1994). Cloning and expression of cDNA of human δ4-3-oxosteroid 5*β*-reductase and substrate specificity of the expressed enzyme. FEBS J..

[52] Coleman R.A., Lewin T.M., Van Horn C.G., Gonzalez-Baró M.R. (2002). Do long-chain acyl-CoA synthetases regulate fatty acid entry into synthetic versus degradative pathways?. J. Nutr..

[53] Bu S.Y., Mashek D.G. (2010). Hepatic long-chain acyl-CoA synthetase 5 mediates fatty acid channeling between anabolic and catabolic pathways. J. Lipid Res..

[54] Li L.O., Ellis J.M., Paich H.A., Wang S., Gong N., Altshuller G., Thresher R.J., Koves T.R., Watkins S.M., Muoio D.M. (2009). Liver-specific loss of long chain acyl-CoA synthetase-1 decreases triacylglycerol synthesis and *β*-oxidation and alters phospholipid fatty acid composition. J. Biol. Chem..

[55] Xu X., Zhao J., Xu Z., Peng B., Huang Q., Arnold E., Ding J. (2004). Structures of human cytosolic NADP-dependent isocitrate dehydrogenase reveal a novel self-regulatory mechanism of activity. J. Biol. Chem..

[56] Berg J.M., Tymoczko J., Stryer L. (2002). Glycolysis is an energy-conversion pathway in many organisms. Biochemistry.

[57] Berg J., Tymoczko J., Stryer L. (2002). The pentose phosphate pathway generates NADPH and synthesizes five-carbon sugars. Biochemistry.

[58] Lu S.C. (2013). Glutathione synthesis. Biochim. Biophys. Acta (BBA)-Gen. Subj..

[59] Adeva-Andany M.M., González-Lucán M., Donapetry-García C., Fernández-Fernández C., Ameneiros-Rodríguez E. (2016). Glycogen metabolism in humans. BBA Clin..

[60] Tang W.H., Martin K.A., Hwa J. (2012). Aldose reductase, oxidative stress, and diabetic mellitus. Front. Pharmacol..

[61] Chelikani P., Fita I., Loewen P. (2004). Diversity of structures and properties among catalases. Cell. Mol. Life Sci..

[62] Liu W., Baker S.S., Baker R.D., Nowak N.J., Zhu L. (2011). Upregulation of hemoglobin expression by oxidative stress in hepatocytes and its implication in nonalcoholic steatohepatitis. PLoS ONE.

[63] Gomer C.J., Ryter S.W., Ferrario A., Rucker N., Wong S., Fisher A.M. (1996). Photodynamic therapy-mediated oxidative stress can induce expression of heat shock proteins. Cancer Res..

[64] Miura Y., Kano M., Abe K., Urano S., Suzuki S., Toda T. (2005). Age-dependent variations of cell response to oxidative stress: Proteomic approach to protein expression and phosphorylation. Electrophoresis.

[65] Eltoweissy M., Müller G.A., Bibi A., Van Nguye P., Dihazi G.H., Müller C.A., Dihazi H. (2011). Proteomics analysis identifies PARK7 as an important player for renal cell resistance and survival under oxidative stress. Mol. Biosyst..

[66] Houstis N.E. (2007). Reactive Oxygen Species Play a Causal Role in Multiple Forms of Insulin Resistance.

[67] Rodrigo R., González J., Paoletto F. (2011). The role of oxidative stress in the pathophysiology of hypertension. Hypertens. Res..

[68] Park E., Giacca A. (2007). Mechanisms underlying fat-induced hepatic insulin resistance. Future Lipidol..

[69] Polimeni L., Del Ben M., Baratta F., Perri L., Albanese F., Pastori D., Violi F., Angelico F. (2015). Oxidative stress: New insights on the association of non-alcoholic fatty liver disease and atherosclerosis. World J. Hepatol..

[70] Kaspar K.L., Park J.S., Brown C.R., Mathison B.D., Navarre D.A., Chew B.P. (2011). Pigmented potato consumption alters oxidative stress and inflammatory damage in men. J. Nutr..

[71] Poudyal H., Panchal S., Brown L. (2010). Comparison of purple carrot juice and *β*-carotene in a high-carbohydrate, high-fat diet-fed rat model of the metabolic syndrome. Br. J. Nutr..

[72] Zhang H., Liu R., Tsao R. (2016). Anthocyanin-rich phenolic extracts of purple root vegetables inhibit pro-inflammatory cytokines induced by H_2_O_2_ and enhance antioxidant enzyme activities in Caco-2 cells. J. Funct. Foods.

[73] Nagle C.A., Klett E.L., Coleman R.A. (2009). Hepatic triacylglycerol accumulation and insulin resistance. J. Lipid Res..

[74] Ahmed M., Neville M.J., Edelmann M.J., Kessler B.M., Karpe F. (2010). Proteomic analysis of human adipose tissue after rosiglitazone treatment shows coordinated changes to promote glucose uptake. Obesity.

[75] Rosenow A., Noben J.-P., Jocken J., Kallendrusch S., Fischer-Posovszky P., Mariman E.C., Renes J. (2012). Resveratrol-induced changes of the human adipocyte secretion profile. J. Proteome Res..

[76] Bouwman F.G., Claessens M., van Baak M.A., Noben J.-P., Wang P., Saris W.H., Mariman E.C. (2009). The physiologic effects of caloric restriction are reflected in the in vivo adipocyte-enriched proteome of overweight/obese subjects. J. Proteome Res..

